# Grafts in tendon repair

**DOI:** 10.1016/j.mtbio.2026.102977

**Published:** 2026-02-28

**Authors:** Miao Zhang, Suzanne M. Mithieux, Ziyu Wang, Anthony S. Weiss

**Affiliations:** a School of Life and Environmental Sciences, University of Sydney, NSW, 2006, Australia; b Charles Perkins Centre, University of Sydney, NSW, 2006, Australia; c The University of Sydney Nano Institute, University of Sydney, NSW, 2006, Australia

**Keywords:** Tendon repair, Graft, Tissue engineering, Scaffold, Biomaterials

## Abstract

Inferior healing after tendon rupture often necessitates the use of grafts to reinforce tendon repair and promote the regeneration of functional tissue. Autograft or allograft implantations are considered the gold standard in tendon reconstruction, though these types of treatment may not always be feasible due to limited availability and patient-specific considerations. In such cases, commercial tendon grafts, encompassing bridging and augmenting grafts are preferred but may not always lead to optimal healing. Consequently, research into the development of more effective constructs for tendon repair is constantly evolving. Here, we review progress in the field by considering the structure and biology of tendons as well as the natural tendon healing processes that inform the evolution of tissue engineered tendon grafts. The limitations of commercial tendon constructs are outlined to establish the opportunities presented by a range of structures, materials, and bioactive adjuncts currently being engineered into tendon grafts to improve tendon recovery and regeneration. We present *in vivo* animal models that have been established to evaluate graft efficacy, and that are critical for the translation to clinical use.

## Graphical abstract

**Figure:**
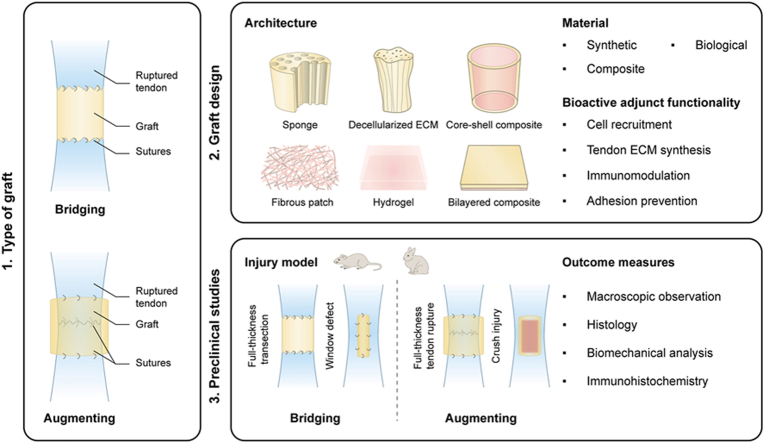

## 1 Introduction

Tendons are soft connective tissues linking muscle and bone. They enable joint movement and stability by transmitting the large-magnitude tensile loads generated by muscles [1]. Tendon tears and ruptures can substantially affect quality of life and represent a growing economic burden on health-care systems. Such tears and ruptures are some of the most frequent musculoskeletal injuries, with the most common injuries occurring in the rotator cuff of the shoulder, the flexor tendons of the hand, and the Achilles tendon at the back of the ankle [2,3]. Of these, the Achilles tendon is the most frequently seen large tendon rupture, with occurrences ranging from 2 to 18 per 100,000 individuals annually [4,5]. Compounding this problem, the incidence of this injury is increasing due to a rise in the number of older adults participating in high-demand sports [2,4,6,7].

The treatment of tendon injuries can involve conservative management or surgical intervention with the optimal regimen remaining unresolved [8]. Conservative treatment commonly utilizes either immobilization with a rigid cast or functional bracing for a few weeks to restore and maintain contact between the two ends of the ruptured tendon, and is then followed by rehabilitation [2,7]. In the case of delayed diagnosis, the success of conservative management may be limited by a lack of apposition of the tendon ends due to scarring and retraction, which necessitates surgical treatments such as primary repair and tendon reconstruction [7,[9], [10], [11]]. Tendon tears are categorized by size, for example full-thickness rotator cuff tears under 1 cm are classified as small, those between 1 and 3 cm are medium, 3 to 5 cm are considered large, and larger than 5 cm are classified as massive [12]. Primary repair is normally performed for tendon defects less than 2-3 cm long [[11], [12], [13], [14], [15]]. Depending on the site of the rupture, primary repair approximates the free tendon end either to the other tendon end using sutures or to the connecting bone using suture anchors or bone screws [12,16].

Tendon reconstruction and augmentation repair strategies are required in the event of a segmental tendon loss that creates an unbridgeable gap, a failed primary repair, or when repair is delayed causing significant peritendinous adhesion and/or tendon retraction [12,[15], [16], [17], [18]]. In a clinical setting, autografts and/or allografts and tendon transfer using neighboring tendons are the gold standard treatments for reconstructing injured tendons as these grafts resemble the original tendon [11,[14], [15], [16], [17],^19^,^20^]. However, their properties and dimensions vary and they have limited availability. Furthermore, due to incompatibility, allografts can induce donor site morbidity and if contaminated, potentially contribute to disease transmission [21,22]. Despite advances in surgical techniques and rehabilitation protocols, inferior healing and peritendinous adhesion persist as major clinical problems following tendon grafting, leading to sub-optimal mechanical strength and impaired motor function and an increased risk of re-rupture [13,[23], [24], [25], [26]]. Disorganized fibrotic scar tissue can arise from the tendon's relatively low cellularity and vascularity [25]. Aberrant cell differentiation towards cartilage and bone after injury can also result in inferior functionality [27]. Peritendinous adhesions form between the tendon and its surrounding sheath, or the sheath and the local tissue [[23], [24], [25],[28], [29], [30]] and are a common complication following injuries to intrasynovial tendons, such as the flexor tendons of the hands and feet [31], but can also be extrasynovial [29,30]. They increase the friction experienced by the tendon during its excursion and gliding motion, contributing to joint stiffness that may require surgical removal of the adhesive tissue [31,32].

The limited availability of tendon grafts and the inferior healing outcomes of current treatments point to the need for implants that bridge tendon defects, restore structural and functional properties and prevent peritendinous adhesion formation. These implants need to provide structural support [[33], [34], [35], [36]], facilitate endogenous cell infiltration and proliferation [[37], [38], [39], [40], [41], [42]], promote tendon extracellular matrix (ECM) deposition [[38], [39], [40],^43^], modulate the inflammatory response [41,[44], [45], [46]], and reduce adhesion formation [44,47]. Depending on the size and severity of the tear, they need to either bridge the gap or act as a patch that can augment the damaged tissue [18,48].

## 2 Tendon biology and healing

### 2.1 Tendon structure and function

#### 2.1.1 Tendon hierarchical structure

**Fig. 1:**
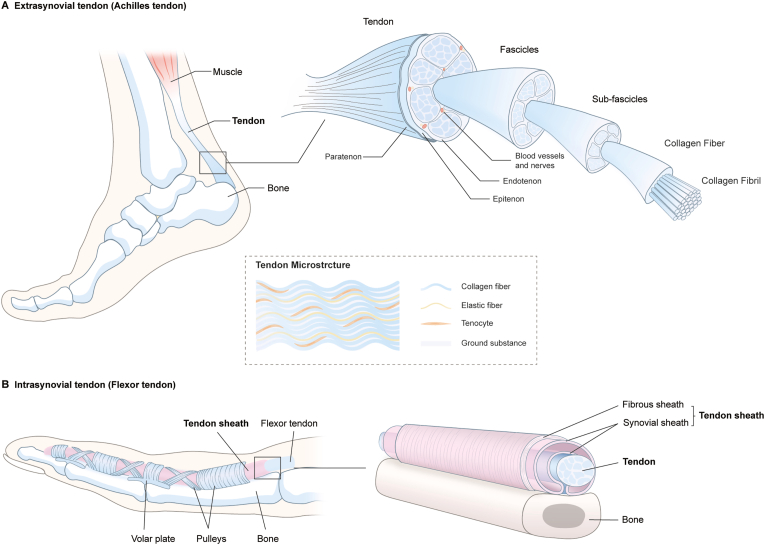
Tendon hierarchical structure. A. Representation of a typical extrasynovial tendon (Achilles tendon) based on Ref. [49]; B. Representation of a typical intrasynovial tendon (finger flexor tendon) showing surrounding peritendinous structures based on Ref. [50,51].

Tendons are hierarchically structured (Fig. 1). Collagen fibrils are grouped into a collagen fiber, several collagen fibers are then bundled and surrounded by a connective tissue called endotenon, to form the subfascicle [49]. A collection of subfascicles forms a fascicle, which is then organized into bundles that comprise the tendon [49]. The endotenon plays a significant role because it binds fiber bundles, carries blood vessels, nerves, and lymphatic vessels to the deeper portion of the tendon [49], and allows the fascicles to slide independently, enabling the tendon to extend, change shape, and transfer tension at varying angles as a joint moves [52,53]. The tendon surface is covered by the epitenon, a dense fibrillar network of collagen that contacts the endotenon on its inner surface [49].

Depending on their surrounding structure, tendons are broadly classified as either extrasynovial (Fig. 1A) or intrasynovial (Fig. 1B) [49]. Intrasynovial tendons, such as flexors tendons in the hand and feet, are contained within a synovial sheath, which forms a closed duct containing peritendinous fluid [49]. Long tendons are usually further covered by a fibrous sheath [49]. The fibrous sheath and synovial sheath, collectively referred to as the tendon sheath, protects the intrasynovial tendon and reduces friction when it glides over bone surfaces and other anatomic structures [[49], [50], [51]]. Extrasynovial tendons that do not have a synovial sheath, like the Achilles tendon, are surrounded by a loose connective tissue called paratenon [49]. The paratenon acts as an elastic envelope to reduce friction between the tendon and the surrounding tissue [49]. The epitenon and paratenon collectively compose the peritenon [54].

#### 2.1.2 Tendon cell population

The tendon cell population is heterogeneous with tenocytes, tendon stem cells, macrophages, endothelial cells, osteoblasts, red blood cells, antigen-presenting cells, and tendon-fibro-adipogenic progenitors [55]. Tenocytes and tendon stem/progenitor cells (TSPCs) are particularly important in tendon repair [27,56]. Tenocytes reside in the tendon core, possess a typical spindle-shaped morphology [57,58] and express tendon-specific genes such as fibromodulin, tenomodulin and thrombospondin 4 [55]. They migrate and deposit new tendon ECM at the repair site following tendon injury [27,56]. TSPCs, also called tendon-derived stem cells (TDSCs), are found throughout the tendon, with subpopulations found in specific locations, including the tendon core [59,60], endotenon [61], paratenon [55], peritenon [61], and tendon sheath [62]. They exhibit multi-potency and can differentiate into tenocytes, adipocytes and chondrocytes [55,59,61,62].

#### 2.1.3 Tendon ECM

The tendon ECM contains collagen and elastic fibers embedded in a tendinous ground substance comprising proteoglycans, glycoproteins, glycosaminoglycans (GAGs), and other small molecules [49,63]. Collagen is the main structural component of the tendon accounting for 65-80% of the dry mass, and 90% is type I collagen [49,64]. The hierarchical organization and rope-like structure of these collagen fibrils contribute to the tendon's ability to resist tension [52,65]. Their wavy crimps absorb shock during tendon stretching at low strain [64] and contribute to the energy-storing capacity of tendon, facilitating extensibility, elasticity, and fatigue resistance [[66], [67], [68]].

Although elastic fibers only comprise 1-2% of the dry mass of the tendon, their value is in their location where they run longitudinally through the tendon [69] and help maintain the collagen crimp through resistance of tensile and transverse shear forces [70,71]. These elastic fibers are highly localized around tenocytes and between the fascicles, suggesting they can also moderate forces experienced by the tenocytes and consequential mechanobiological responses by cells to load [69]. Moreover, the enrichment of elastic fibers in the interfascicular space likely provides the tendon sheath with the benefits of elastic recoil and resilience [69].

Proteoglycans intersperse the collagenous units at the fibril, fiber, and fascicle levels of the tendon hierarchy [72]. Among them, decorin and biglycan have been used as tendon-specific markers in research studies [35,42,[73], [74], [75], [76], [77]]. Decorin is a small leucine-rich proteoglycan that envelops the collagen fibrils [64,72] and controls their diameter by preventing lateral fusion [64,72,78]. Decorin also plays a role in promoting collagen fibrillar slippage and improving lateral interactions during tensile deformation, thus maximizing the load before failure occurs [64,72]. Biglycan guides the formation and maintenance of mature collagen fibrils by regulating the self-renewal and differentiation of TSPCs [59] and preventing local ossification [59,72].

ECM glycoproteins, such as cartilage oligomeric matrix protein (COMP) [67,79], tenomodulin [80,81], and tenascin-C [82] are important in tendon development and healing. COMP is the most abundant glycoprotein in tendons where it mediates tendon growth and contributes to the mechanical strength of tendons [67,79]. Tenomodulin is essential for collagen fibril maturation and has been widely used as a mature tendon cell marker [80,83] where it is a positive regulator of TSPC migration, proliferation and self-renewal [81]. During early tendon healing, tenomodulin can prevent the differentiation of TSPCs into adipocytes, which is a common pathological change that occurs in ruptured tendons [81], and modulate inflammatory responses to further facilitate tendon healing [81]. Tenascin-C participates in tenogenesis and may contribute to tissue elasticity [67,82].

### 2.2 Natural tendon healing in adults

Natural tendon healing generally progresses through inflammatory, proliferative, and remodeling stages [63]. The inflammatory stage typically lasts about a week, during which time the wound site is infiltrated by fibroblasts and immune cells including monocytes, neutrophils, and macrophages [84]. Fibroblasts recruited to the site begin to synthesize various components of the ECM, which help to connect the ruptured tendon ends and partially stabilize the structure [23,63]. Transient inflammation is required for tissue repair, while extreme or persistent inflammation can lead to excessive fibrosis, resulting in suboptimal healing [24]. The proliferative stage of healing begins roughly two days into the injury response and spans 1-3 weeks [85]. This stage is characterized by continued fibroblast recruitment with rapid proliferation [24,63,86]. These fibroblasts continue to deposit a highly disorganized fibroblastic tissue comprising type III collagen to bridge the ruptured tendon [87,88]. The remodeling stage begins at 6-8 weeks after injury and can continue for years [23], during which time the disorganized type III collagen matrix is gradually replaced by aligned and mechanically resistant type I collagen [88]. The remodeling phase is associated with a decrease in tendon adhesions and a return to normal tendon function [88].

From a cellular perspective, tendon healing occurs both intrinsically and extrinsically [27,89]. Intrinsic healing involves the proliferation of cells that have migrated from the tendon core to the injured region. These cells express higher amounts of scleraxis [27,88,89], which is a transcription factor [90] that directs the formation of an organized ECM enriched in type I collagen [89,91]. Intrinsic healing dominates tendon healing in neonates and regenerative organisms such as zebrafish, resulting in the regeneration of functional tendon tissue with minimal scar and adhesion tissue [27,56]. However, intrinsic cells have very little capacity for self-repair in adult human tendons, leading to extrinsic healing [27,92]. Extrinsic healing involves the proliferation of cells originating in structures surrounding the tendon core and external tissue [27,89]. These cells proliferate more rapidly and exhibit higher transition potential toward a myofibroblastic phenotype compared to intrinsic cells. This can result in the accumulation of myofibroblasts that contribute to the formation of a highly disorganized scar [27,55] comprised of type I and type III collagen that persistently impairs the biomechanical function of the repaired tendon [27,87,88]. The scar tissue can also bridge between the tendon and its surrounding tissues, resulting in peritendinous adhesions [93]. Beneficially however, extrinsic tissues also contain TSPCs that can differentiate into tenocytes [55,[60], [61], [62],^94^] and secrete stimulatory factors to intrinsic cells [91], which can be utilized to promote tendon repair.

In addition to tenocytes and TSPCs, macrophages help to heal tendon through phenotypic polarization in response to changes in their environment, leading to enrichment for M1 and M2 subtypes [95,96]. M1 macrophages clear pathogens and release proinflammatory cytokines [97]. In contrast, M2 macrophages modulate inflammatory responses, remove debris, and facilitate angiogenesis, tissue repair and remodeling [97]. As inflammation progresses, the M1 population is gradually supplanted by M2 macrophages [98]. Strategies that promote macrophage polarization towards the M2 phenotype and suppress M1 polarization in the early phase of tendon healing correlate with increased collagen deposition and maturation and result in a uniform distribution of dense collagen fibrils and improved biomechanical properties [37,39,99,100]. However, the underlying mechanism of this phenomenon and which specific M2 subpopulations are involved is unclear. Further studies are needed to identify these subsets of macrophages. Recent studies have identified two further subtypes of macrophages implicated in tendon regeneration and scar formation: secreted phosphoprotein 1-expressing (SPP1^+^) macrophages and folate receptor beta-expressing (FOLR2^+^) macrophages [101,102]. SPP1^+^ macrophages are found in tendon adhesion tissue and exhibit a profibrotic phenotype by promoting myofibroblast differentiation and collagen deposition [101,102]. FOLR2^+^ macrophages exhibit an antifibrotic phenotype that limits excessive fibrosis in the human tendon adhesion process [101], and present novel cellular targets for peritendinous adhesion treatment.

## 3 Graft-assisted tendon healing – what is commercially available?

Tendon grafts enhance the clinical outcomes of tendon healing and/or reconstruct a tendon tear that is otherwise irreparable. These grafts are broadly categorized as either bridging or augmenting grafts. Bridging tendon grafts connect the ends of fully or partially ruptured tendons, or bridge between a tendon and the connecting bone, which is otherwise unable to reapproximate [103,104]. They provide mechanical support during the repair process and may either remain or gradually degrade to be replaced by a newly formed tendon matrix. In contrast, augmenting tendon grafts are applied over a ruptured tendon that has either undergone primary repair [105,106] or is bridged with another graft [[107], [108], [109]], including through tendon transfer. They can be administered by overlaying or circumferentially wrapping the injury site and secured in place with suturing. For example, for reinforcement of rotator cuff and patellar tendon tears, one extremity of the augmenting graft is sutured to the tendon ends while the opposing extremity is sutured to the osseous insertion site after a primary repair [[110], [111], [112]] but these grafts are not intended to provide full mechanical support; instead they facilitate tendon healing and thereby reduce the re-tear rate of the injured tendon and so improve clinical outcomes [18,48,113]. They also act as barriers between the injured tendon and the surrounding tissues to limit the formation of peritendinous adhesions [[114], [115], [116], [117]].

Allografts, xenografts, and synthetic, biological, and hybrid grafts, have been developed and commercialized to assist tendon healing, with most used as augmenting grafts (Table 1). Out of 36 commercially available products in Table 1, 17 have clinical reports (Table 2), as clinical testing is nonmandatory if a graft is proven to be substantially equivalent to a predicate device [184]. Clinical scores are based on pain level, mobility including flexion, rotation, and abduction, muscle strength, mental health, and time to return to work. While most of these grafts are able to assist and improve tendon repair, problems such as moderate to high re-tear rates, mild to moderate pain, restricted mobility, and muscle weakness are common.

**Table 1:** Commercially available grafts for tendon repair.

Structure	Tendon device	Manufacturers	Material	Clinical outcomes
Decellularized Tendon Allograft	QuickGraft™ Pre-Sutured Allograft Tendons [118]	MTF Biologics	Human tendon; UHMWPE sutures	NA
Decellularized allograft	ArthroFLEX® Decellularized Dermal Allograft [119]	Arthrex, Inc.	Human acellular dermis matrix	Used as an augmenting graft;
				Reduced pain level; Improved functionality of torn rotator cuff;
				Retear reported.
	GraftJacket™ [120]	Wright Medical	Human dermal matrix	Used as an augmenting graft and bridging graft;
				Reduced pain level; Improved functionality;
				Retear, partial retear, reported;
				Persistent mild pain reported.
Decellularized xenograft	TissueMend™ [121]	TEI Bioscience Inc	Bovine dermis matrix	NA
	ProPatch® Soft Tissue Repair Matrix [122]	CryoLife, Inc.	Bovine pericardium matrix	NA
	OrthADAPT® Bioimplant [123]	Synovis Orthopedic and Woundcare, Inc.	Equine pericardium matrix (crosslinked)	Used as an augmenting graft;
				High retear rate;
				Caused adverse events.
	Medeor™ Matrix [124]	Kensey Nash Corporation	Porcine dermis matrix	NA
	Zimmer® Collagen Repair Patch[125]	Zimmer Biomet	Porcine dermis matrix	NA
	TRELLIS™ Collagen Ribbon [113,126]	Wright Medical Technology, Inc.	Porcine dermis matrix (woven)	NA
	Miromatrix Biological mesh TW [127,128]	Miromatrix Medical Inc.	Porcine liver matrix	NA
	CuffPatch™ [129]	Arthrotek	Porcine small intestinal submucosa matrix	NA
	Restore™ [129]	DePuy Synthes	Porcine small intestinal submucosa matrix	NA
Anisotropic fibrous patch	BioBridge® Collagen Matrix [130]	Fibralign Corporation	Porcine collagen	NA
	TAPESTRY® Biointegrative Implant [131]	Embody, Inc.	Type I collagen; PDLLA	Used as an augmenting graft;
				Degraded within 6 months post-implantation;
				Partial retear reported.
Fibrous patch	Rotium™ Bioresorbable Wick [132]	Nanofiber Solutions, LLC	PLCL, PGA	Used as bridging patch between tendon and bone;
				Improved functionality;
				Degraded within 10.5 month of implantation;
				Retear can occur.
	SpinMedix® Absorbable Fibrous Membrane [133]	CelestRay Biotech company, LLC	PLGA; PLA-b-PEG	NA
	Permacol™ [134]	Tissue Science Laboratories PLC	Porcine dermal collagen (crosslinked), elastin fibers	More commonly used to repair abdominal wall.
Braided scaffold	STR GRAFT [135]	Soft Tissue Regeneration, Inc.	PLLA	NA
Knitted scaffold	FlexBand®/FlexPatch®/FlexBand® Plus [136]	International Life Sciences	ARTELON®	Used as a bridging graft and an augmenting wrap for Achilles tendon;
				Reduced pain level; Improved functionality;
				No retear reported.
	LARS™ Ligaments [137]	Corin group	PET	Used as a bridging graft and an augmenting wrap;
				Improved functionality;
				Pain level not efficiently compared;
				No retear reported.
	Mersilene™ Polyester Fiber Mesh[138]	Ethicon	PET	Used as a bridging graft;
				Improved functionality;
				Retear reported;
				Pain reported.
	SportMesh™ Artelon® Tissue Reinforcement [139]	Artimplant AB	ARTELON®	NA
	Pitch-Patch Tissue Reinforcement Device [140]	Xiros, Ltd	PET	Used as an augmenting graft;
				Reduced pain level; Improved functionality;
				High retear rate;
				Revision surgery required occasionally.
	Integrity™ Implant [141]	Anika Therapeutics, Inc.	Resorbable Hyaff fibers and non-resorbable PET	NA
Woven tape	Poly-Tape/Infinity-Lock Soft Tissue Reinforcement Device [142]	Xiros, Ltd	PET	Used as a bridging or an augmenting graft;
				No retear reported;
				Persistent mild pain reported;
				Limited clinical evidence (3 cases).
Woven patch	X-Repair [143]	Synthasome, Inc.	PLLA	Used as an augmenting graft;
				Improved functionality;
				Retear reported.
Sheet or Hydrogel	VersaWrap® [116]	Alafair Biosciences, Inc.	Alginate; Hyaluronic acid	Used as an augmenting wrap;
				Reduced peritendinous adhesion.
Sheet	BioBlanket™ Surgical Mesh [144]	Kensey Nash Corporation	Bovine collagen	NA
	Collagen Tendon Sheet-D[145]	Rotation Medical, Inc.	Type I collagen matrix	Used as an augmenting graft;
				Reduced tendon tear size in 94% of patients;
				Reduced pain level; Improved functionality;
				Post-operative complications such as scapular dyskinesia requiring prolonged therapy and bracing were reported.
	OrthoWrap® Bioresorbable Sheet [117]	MAST Biosurgery, Inc	PLLA	Used as an augmenting wrap;
				Improved extension lag of the metacarpophalangeal joint compared to the standard repair protocol.
	Regeneten Bioinductive Implant [146][147]	Smith and Nephew, Inc	Bovine type I collagen	Used as an augmenting graft;
				Reduced pain level; Improved functionality;
				High patient satisfaction;
				Revision surgery required occasionally due to retear or failure and complications.
	Seprafilm® Bioresorbable Membrane [114]	Genzyme Corporation	Sodium hyaluronate; carboxymethyl cellulose	More commonly used to prevent adhesion after abdominal surgery.
	TenoMend™ Collagen Tendon Wrap [115]	Collagen Matrix Inc	Bovine type I collagen	Used as an augmenting graft;
				Reduced tear size;
				Reduced pain level; Improved functionality;
				Excessive swelling and significant pain reported occasionally.
	Tendon Wrap™ Tendon Protector [148]	Integra LifeSciences Corporation	Bovine type I collagen and GAG (crosslinked)	NA
Fiber-reinforced porous sheet	BioBrace™ [105,149,150]	Biorez	Collagen; PLLA microfilaments	Used as an augmenting graft;
				Improved strength and mobility of the injured joint;
				Integrated well with the underlying tendon after 6 months;
				Limited clinical evidence (2 cases).
Bilayered membrane	Nexo-Gide™ Bilayer collagen membrane [151]	Geistlich Pharma AG	Porcine type I and III collagen	NA

**Abbreviation**: GAG – glycosaminoglycancan; PET - polyethylene terephthalate; PDLLA – poly(D,L-lactide); PGA – poly(glycolic acid); PLCL – poly(L-lactic acid-co-ε-caprolactone); PLA-b-PEG – poly(ethylene glycol)-b-poly(lactic acid); PLGA – poly(lactic-co-glycolic) acid; PLLA – poly(L-lactide-co-D,L-lactide); NA – not available; UHMWPE – ultrahigh molecular weight polyethylene.

**Table 2:** Clinical studies on commercially available tendon repair grafts.

Study type	Year	Injury type	Tendon involved	Grafting technique	Cases (n)	Follow-up (months)	Re-tear/failure to heal rate	Clinical Outcomes	Complications/procedure failure	Ref
Allograft										
ArthroFLEX® Decellularized Dermal Allograft										
Prospective Comparative	2015	Large to massive tear	Rotator cuff	Augmenting patch	15 control; 20 graft	22-26	10.4 %	Patient-reported Outcome Measures:	1 superficial skin infection.	119
								Mean pain level: from 6.9 to 4.1 (control) vs from 6.8 to 0.9 (graft) (p = 0.024);		
								ASES score: from 62.1 to 72.6 (control) vs from 63.8 to 88.9 (graft) (p = 0.02);		
								SF-12 PCS improvement: 6.1 (control) vs 11.8 (graft) (p = 0.052);		
								SF-12 MCS improvement: 3.7 (control) vs 5.1 (graft) (p = 0.03);		
								WORC index improvement: 13% (control) vs 38% (graft) (p = 0.0412).		
Therapeutic	2016	Massive retears	Rotator cuff	Augmenting patch	13	24	7.7%	Patient-reported Outcome Measures:	None	152
								ASES total score: 64.5 (pre-op) vs 86.0 (post-op) (p = 0.094);		
								SF-12 PCS: 44.5 (pre-op) vs 52.9 (post-op) (p = 0.005);		
								SANE score: 54.3 (pre-op) vs 74.8 (post-op) (p = 0.011);		
								QuickDASH score: 36.5 (pre-op) vs 11.3 (post-op) (p = 0.006).		
**GraftJacket™**										
Prospective	2007-2011	Irreparable tear	Rotator cuff	Bridging	45	84-150	0%	Patient-reported Outcome Measures:	None	153
								Pain score: 6.1 (pre-op) vs 2.1 (9.1-year post-op);		
								OSS score: 24.7 (pre-op) vs 42.0 (1-year post-op) vs 42.8 (9.1 year post-op) (p < 0.001).		
Prospective	2007-2008	Massive tear	Rotator cuff	Bridging	24	29-40	30%	Patient-reported Outcome Measures:	1 partial re-tear because the patient began weightlifting and swimming at 2 weeks postoperatively.	104
								VAS Pain score: 5.4 (pre-op) vs 0.9 (post-op) (p = 0.0002);		
								ASES score: 66.6 (pre-op) vs 88.7 (post-op) (p = 0.0003);		
								SF-12: 48.8 (pre-op) vs 56.8 (post-op) (p = 0.03);		
								ROM Outcome Measures:		
								Forward flexion: 111.7° (pre-op) vs 157.3° (post-op) (p = 0.0002);		
								Abduction: 105.0° (pre-op) vs 151.7° (post-op) (p = 0.0001);		
								External rotation: 46.2° (pre-op) vs 65.1° (post-op) (p = 0.001);		
								Internal rotation: Sacrum (pre-op) vs upper lumbar (post-op);		
								Strength Outcome Measures:		
								Supraspinatus: 7.2 (pre-op) vs 9.4 (post-op) (p = 0.0003);		
								External rotation: 7.8 (pre-op) vs 9.3 (post-op) (p = 0.002);		
								Ultrasonography:		
								74% fully intact repair, 26% partially intact repair with a defect detected in either the graft-tendon interface or graft-humerus interface.		
Prospective	2007-2011	Large to massive tear	Rotator cuff	Bridging	61	12-72	3% partial retears	Patient-reported Outcome Measures:	1 persistent pain;	154
								Pain score: 7 (pre-op) vs 0.8 (1-year post-op);	1 deep wound infection.	
								OSS score: 26 (pre-op) vs 42 (1-year post-op) (p = 0.001);		
								ROM Outcome Measures:		
								Forward flexion: 97° (pre-op) vs 160° (post-op) (p = 0.001);		
								Abduction: 90° (pre-op) vs 155° (post-op) (p = 0.001);		
								External rotation: 42° (pre-op) vs 60° (post-op) (p = 0.04);		
								Internal rotation: Sacrum (pre-op) vs upper lumbar (post-op);		
								Strength Outcome Measures:		
								Abduction: 4 (pre-op) vs 5 (post-op) (p = 0.01);		
								External rotation: 4 (pre-op) vs 5 (post-op) (p = 0.01);		
								Internal rotation: 5 (pre-op) vs 5 (post-op) (p = 0.04).		
Prospective	2009-2012	Large tear	Rotator cuff	Bridging	14	23-59	7%	Patient-reported and Physician Assessment Outcome Measures:	1 large retear with fibrosed margins.	155
								CMS score: 83 (post-op) vs 87.2 (non-operated side) (p = 0.03);		
								Patient-reported Outcome Measures:		
								QuickDASH score: 5.4 (post-op) vs 4.7 (non-operated side) (p = 0.6);		
								OSS score: 46 (post-op) vs 46.2 (non-operated side) (p = 0.92);		
								Strength outcomes:		
								Abduction: 11.4 Ib (post-op) vs 15.5 Ib (non-operated side).		
Retrospective	2013	Massive tear	Rotator cuff	Bridging	14	18-52	0%	Patient-reported Outcome Measures:	None	156
								VAS score: 7.4 (pre-op) vs 1.7 (post-op) (p = 0.001);		
								ASES score: 23.8 (pre-op) vs 72.3 (post-op) (p = 0.001);		
								ROM Outcome Measures:		
								Forward flexion: 73.6° (pre-op) vs 129.3° (post-op) (p = 0.002);		
								Abduction: 67.5° (pre-op) vs 117.9° (post-op) (p = 0.002);		
								External rotation: 7.9° (pre-op) vs 43.2° (post-op) (p = 0.001);		
								Strength Outcome Measures:		
								Forward flexion: 5/5 (21% patient), 4/5 (36% patient), 3/5 (21% patient), 0/5 (21% patient);		
								Abduction: 5/5 (7% patient), 4/5 (50% patient), 3/5 (21% patient), 0/5 (21% patient);		
								External rotation: 5/5 (7% patient), 4/5 (43% patient), 3/5 (29% patient), 0/5 (21% patient).		
Prospective	2015-2016	Massive tear	Rotator cuff	Bridging	2 (single layered); 18 (double layered)	12	0%	Patient-reported Outcome Measures:	1 postoperative adhesive capsulitis;	157
								SF-12 PCS: 47.4 of 100;	1 developed persistent pain after surgery but no evidence of infection or rejection.	
								SF-12 MCS: 56.6 of 100;		
								OSS score (single layered group): 12.5 (24.5 months post-op);		
								OSS score (double layered group): 45.4 (17 months post-op);		
								Recovery time:		
								Operative recovery to maximum function: 5 months;		
								Returned to work in 16 weeks: 87.5%;		
								Returned to driving in 8 weeks: 93.8%;		
								Patient satisfaction: 90%		
Prospective	2004-2007	Massive tear	Rotator cuff	Augmenting patch	45	24-68	0%	Patient-reported Outcome Measures:	1 deep wound infection in an immunocompromised patient.	120
								WORC score: 75.2;		
								ASES score: 84.1.		
								Patient-reported Outcome Measures and Physician Assessment:		
								UCLA Shoulder score: 18.4 (pre-op) vs 27.5 (post-op) (p < 0.000).		
Prospective	2012	Large tear	Rotator cuff	Augmenting patch	20 (control);	12-38	60% (control);	Patient-reported Outcome Measures and Physician Assessment:	Control group:	158
					22 (graft)		15% (graft)	UCLA Shoulder score: from 15.9 to 28.3 (control) vs from 13.3 to 28.2 (graft) (p = 0.43);	2 cellulitis; 1 shoulder bursitis; 1 post-traumatic fibrosis	
								CMS score: from 45.8 to 85.3 (control) vs from 41.0 to 91.9 (graft) (p = 0.008);	Graft group:	
								Patient-reported Outcome Measures:	1 recurrent shoulder bursitis.	
								ASES score: from 46.0 to 94.8 (control) vs from 48.5 to 98.9 (graft) (p = 0.035);		
								MRI:		
								Tear size: 40% intact (control) vs 85% intact (graft) (p < 0.01).		
**Xenografts**										
**OrthADAPT® Bioimplant**										
Prospective	2011-2012	Complete rupture	Rotator cuff	Augmenting patch with BM-MSCs	13	12	60% in graft group;	The study was stopped when the third adverse event was detected.	4 patients (1 from graft group and 3 from graft + cell group) had supraclavicular cysts and developed subacromial inflammatory tissue;	159
							62.5 % in graft/cell group		3 patients had supraclavicular ganglions.	
**Synthetic grafts**										
**FlexBand®/FlexPatch®/FlexBand® Plus**										
Retrospective	2018-2020	Insertional tendinosis	Achilles	Bridging	18	3 to 11	0%	Patient-reported Outcome Measures:	1 wound breakdown; 1 suture anchor pull-out from the calcaneus.	160
								Mean VAS pain score: 6.19 (pre-op) vs 0.83 (post-op) (p < 0.01);		
								Strength Outcome Measures:		
								Plantarflexion: 5/5 (83.24% patient), 4/5 (11.76% patient);		
								ROM Outcome Measures:		
								Dorsiflexion: >10° (94.44% patient);		
								Patient satisfaction: 93.33% (14 of 15).		
Retrospective	2021-2023	Chronic rupture	Achilles	Bridging	7	24-36	0%	Patient-reported and Physician-assessed Outcome Measures:	1 superficial post-operative infection; 2 postoperative numbness in sural nerve territory;	161
								AOFAS score: 59 (pre-op) vs 91 (post-op) (p = 0.018);	1 sensitive scar with paraesthesia that settled by 12 months postoperatively	
								ATRS score: 92 of 100;	2 occasional cramps in calf muscles on the operated side.	
								VAS pain score: 0 of 10;		
								VAS function score: 8 of 10;		
								Recovery time:		
								Average time off work: 14 weeks.		
Case report	2022	Chronic rupture	Achilles	Bridging along with FHL tendon transfer	1	12	0%	Patient-reported Outcome Measures:	None	162
								PROMIS Global Physical Health T-score: 42 (pre-op) vs 54 (post-op)		
								Foot Function Index: 100 (pre-op) vs 21 (post-op);		
								PROMIS Pain T-Score: 71 (pre-op) vs 53 (post-op);		
								Strength Outcome Measures:		
								Plantar flexion: 5/5;		
								ROM Outcome Measures:		
								Plantar flexion: 40°;		
								Dorsiflexion: 5°;		
								MRI:		
								Complete healing of the graft at the muscle and bone attachments.		
Case report	2023	Tendon laceration	Extensor hallicus longus tendon	Augmenting wrap (after allograft bridging)	1	3	0%	Strength Outcome Measures:	Numbness and stiffness were present, but was gradually improving	107
								Hallux dorsiflexion: 4/5;		
								ROM Outcome Measures:		
								Hallux dorsiflexion: 60°;		
								Plantar flexion: 10°.		
**LARS ligament**										
Single-centre retrospective	2007-2016	Rupture after Total knee arthroplasty	Patellar tendon	Augmenting patch	6	24-60	0%	Patient-reported and Physician-assessed Outcome Measures:	1 superficial infection	163
								Mean KSS knee score: 63.3;		
								Mean KSS functional score: 35;		
								ROM Outcome Measures:		
								Extensor lag: <10° (4 knees), >20° (2 knees in 1 patient);		
								Radiography and ultrasonography:		
								Mean IS-I: 1.16;		
								Mean patellar tendon thickness increase: 127.12%;		
								1 knee presented radiolucent lines around tibial stem without any symptom;		
								Ambulatory ability:		
								2 patients were able to walk independently; 2 patients needed one crutch to walk; 1 patient was unable to walk without a therapist.		
Retrospective	2009-2020	Rupture	Quadriceps tendon and patellar tendon	Augmenting patch	10	3-132	0%	Patient-reported Outcome Measures:	None	164
								Lysholm score: 74.2;		
								ROM Outcome Measures:		
								Flexion: 117°;		
								Extension lag: 18°.		
Case series	2013-2018	Chronic rupture	Quadriceps tendon	Augmenting patch	6	1.5	0%	ROM Outcome Measures:	1 superficial wound infection.	106
								Full range of movement: 4 patients (66%);		
								Full range of flexion but 10° extensor lag: 1 patient (17%);		
								5-100° flexion: 1 patient (17%);		
								Strength Outcome Measures:		
								MRC grade strength: 5/5.		
Case report	2021	Chronic rupture	Quadriceps tendon	Augmenting patch with hamstring autograft bridging	1	6	0%	Patient-reported Outcome Measures:	None	108
								VAS pain score: 0;		
								ROM Outcome Measures:		
								Flexion: >100°;		
								Extension lag: 10-15°.		
Case report	2023	Fibroma of tendon sheath	Quadriceps tendon	Bridging	1	20	0%	Strength Outcome Measures:	None	165
								Quadricep muscle strength: 4/5 (8-week post-op);		
								Recovery time:		
								6-month post-op: patient return to daily living activities, engaging in normal squatting, cycling, swimming and other low-energy exercise;		
								12-month post-op: patient can play confrontational sports like basketball;		
								20-month post-op: patient recovered well and had no discomfort.		
**Mersilene mesh**										
Prospective	1996-2002	Massive full-thickness tear	Rotator cuff	Bridging	41	43	9%	Patient-reported and Physician-assessed Outcome Measures:	1 torn upper layers of mesh; 3 partial retear between graft and musculotendinous unit; 11 (26%) patients still suffer from pain postoperatively	138
								CMS scores: 25.7 (pre-op) vs 72.1 (post-op) (p < 0.001).		
**OrthoWrap® Bioresorbable Sheet**										
Prospective	2009-2011	Rupture	Hand extensor tendon	Augmenting wrap	43 (control);	3	0%	Patient-reported Outcome Measures:	None	117
					42 (graft)			VAS pain score: 2.4 (control) vs 2.1 (graft) (p = 0.19);		
								ROM Outcome Measures:		
								Extension lag: 14.79° (control) vs 12.29° (graft) (p = 0.001);		
								Meracarpophalangeal joint flexion: 68.11° (control) vs 71.42° (graft) (p = 0.06);		
								Strickland classification: 13.2% good, 86.8% fair in control group; 34.2% good, 65.8% fair in graft group (p = 0.03).		
**Pitch-Patch Tissue Reinforcement Device**										
Prospective	2020	Massive tear	Rotator cuff	Augmenting patch	50	52	14%	Patient-reported and Physician-assessed Outcome Measures:	8 patients required revision surgery: 6 frozen shoulder or arthrofibrosis; 1 re-rupture and 1 crepitus.	166
								CMS score: 36.5 (pre-op) vs 83.4 (post-op) (p < 0.0001);		
								Patient-reported Outcome Measures:		
								SSV score: 40.3 (pre-op) vs 89.6 (post-op) (p < 0.0001).		
Retrospective case	2023	Massive tear	Rotator cuff	Augmenting patch	15	24	53%	Patient-reported and Physician-assessed Outcome Measures:	3 patients showed Sugaya grade 4 retear; 5 patients showed Sugaya grade 5 retear; 3 of these 8 patients showed an increase in fatty infiltration.	167
								CMS scores: 33 (pre-op) vs 81 (post-op) (p = 0.03);		
								Patient-reported Outcome Measures:		
								VAS pain score: 5.3 (pre-op) vs 0.2 (post-op) (p < 0.01);		
								SSV score: 34.7 (pre-op) vs 89.5 (post-op) (p = 0.007);		
								ROM Outcome Measures:		
								Forward flexion: 111° (pre-op) vs 163° (post-op) (p = 0.004);		
								External rotation: 37° (pre-op) vs 38° (post-op) (p = 0.5);		
								Strength Outcome Measures:		
								Abduction force: 0.7 kg (pre-op) vs 6.5 kg (post-op) (p < 0.01).		
**Poly-Tape/Leeds-Keio™ graft/Neoligaments©**										
Case report	2020	Spontaneous rupture	Quadriceps	Bridging	2	12	0%	Patient-reported Outcome Measures:	None	103
								Lysholm Knee Scoring Scale: 84% and 82%;		
								IKDC score: 67.8% and 50.6 %;		
								VAS score: 2 and 4;		
								ROM Outcome Measures:		
								Both patients recovered the full extension and a 120° pain-free flexion of the knee;		
								MRI:		
								Complete, bilateral, bio-integration of the augmentation patch for each knee.		
Case report	2021	Chronic rupture	Quadriceps	Augmenting patch (along with Achilles tendon allograft augmentation)	1	18	0%	ROM Outcome Measures:	Psoriasis originated from patient's left leg and spread to his groin, lower back and the right leg. However, it is difficult to attribute or exclude directly the allogenic graft reaction or Poly-Tape hypersensitivity.	109
								Extension: recovered (same as the contralateral leg);		
								Leg flexion: 130°;		
								Patient was satisfied.		
Retrospective	2022-2025	3 chronic rupture; 2 acute rupture	Patella or quadriceps tendon	Bridging or augmenting; A decellularized dermal patch was applied to augment the repair in one case.	5	38	0%	ROM Outcome Measures:	None	168
								Extension lag: 10°, 0°, 10°, 3°, 10°;		
								Flexion range: 120°, 90°, 90°, 90°, 90°;		
								Ambulatory ability:		
								4 patients able to mobilize independently without any walking aid; 1 patient able to mobilize independently with a single stick.		
**Rotium™ Bioresorbable Wick**										
Case series	2019-2020	Small- and medium sized tear	Rotator cuff	Bridging	33	8.6	9%	Patient-reported Outcome Measures:	1 transtendon failure medial to the tendon-bone interface; 1 failure occurred at the subscapularis insertion site; 1 anchor pullout.	169
								ASES score: 36.9 (pre-op) vs 86.8 (post-op) (p < 0.00001);		
								SST score: 4.2 (pre-op) vs 11.7 (post-op) (p < 0.00001);		
								Active ROM Outcome Measures:		
								Mean forward flexion: 90° (pre-op) vs 155° (post-op) (p < 0.001);		
								Mean shoulder abduction: 80° (pre-op) vs 145° (post-op) (p < 0.001);		
								Mean external rotation with the arm in neutral position: 58° (pre-op) vs 66° (post-op) (p < 0.01);		
								Mean external rotation with the shoulder at 90° of abduction: 69° (pre-op) vs 83° (post-op) (p < 0.001);		
								Mean internal rotation with the shoulder at 90° of abduction: 50° (pre-op) vs 63° (post-op) (p = 0.004);		
								MRI:		
								Healing of the repaired tendon in 91% of patients.		
								Graft not visible, suggesting resorption in 10.5 months post-op (range, 6-16.7 months).		
**X-Repair**										
Case series	2014	Massive tear	Rotator cuff	Augmenting patch	18	42	22%	Patient-reported Outcome Measures:	None	170
								ASES score: 25 (pre-op) vs 71 (post-op) (p < 0.05);		
								MRI and ultrasonography:		
								78% intact rotator cuff repair 42-month post-op.		
**Biological grafts**										
**Collagen Tendon Sheet-D**										
Prospective multicenter	2017	Partial-thickness tear	Rotator cuff	Augmenting patch	33	12	0%	Patient-reported and Physician-assessed Outcome Measures:	1 showed increased accumulation of prominent subacromial fluid without clinical symptoms	171
								CMS score: from 57.1 to 81.4 (p < 0.0001);		
								Patient-reported Outcome Measures:		
								ASES pain score: from 4.2 to 0.6 (p < 0.0001)		
								ASES shoulder function score: from 16.9 to 25.1 (p < 0.0001)		
								ASES shoulder index score: from 57.0 to 89.1 (p < 0.0001)		
								MRI:		
								24% completely filled in; 70% reduction in defect size of at least 1 grade; 3 % remained unchanged.		
Case series	2014-2015	Full-thickness large or massive tear	Rotator cuff	Augmenting patch	23	24	9%	Patient-reported Outcome Measures:	1 failed to heal the supraspinatus tendon; 1 had pain and dysfunction due to progression of arthritis and further atrophy of the rotator cuff muscle that required reverse total shoulder arthroplasty;	172
								ASES score: 82.87;	8 (35%) had postoperative scapular dyskinesia requiring prolonged therapy and bracing.	
								MRI and ultrasonography:		
								Thickness of Rotator Cuff: 5.13 mm.		
**Regeneten Bioinductive Implant**										
Retrospective case	2019	Partial-thickness and full-thickness tear	Rotator cuff	Augmenting patch	173	12	2%	Patient-reported Outcome Measures:	4 failed to repair; 1 post-operative infection; 1 deep vein thrombosis and adhesive capsulitis; 1 postoperative stiffness; 1 recurrent effusion.	173
								Partial-thicness tear (n = 90)		
								VAS pain score: 5.3 (pre-op) vs 1.1 (post-op) (p < 0.001);		
								SANE score:42.5 (pre-op) vs 86.0 (post-op) (p < 0.001);		
								VR-12 PCS score:35.8 (pre-op) vs 49.7 (post-op) (p < 0.001);		
								ASES function score:14.2 (pre-op) vs 24.8 (post-op) (p < 0.001);		
								ASES shoulder score:47.0 (pre-op) vs 85.6 (post-op) (p < 0.001);		
								WORC score: 38.2 (pre-op) vs 84.4 (post-op) (p < 0.001)		
								Full-thickness tear (n = 83)		
								VAS pain score: 5.2 (pre-op) vs 1.3 (post-op) (p < 0.001);		
								SANE score:39.2 (pre-op) vs 80.7 (post-op) (p < 0.001);		
								VR-12 PCS score:34.5 (pre-op) vs 45.7 (post-op) (p < 0.001);		
								ASES function score:13.1 (pre-op) vs 24.0 (post-op) (p < 0.001);		
								ASES shoulder score: 45.5 (pre-op) vs 83.8 (post-op) (p < 0.001);		
								WORC score: 35.0 (pre-op) vs 80.1 (post-op) (p < 0.001)		
Case report	2019	Partial-thickness tear	Patellar	Augmenting patch with injection of platelet-rich plasma	1	10	0%	Patient-reported Outcome Measures:	None	174
								VAS score: 7 (pre-op) vs 2 (post-op);		
								MRI:		
								Intact repair at 10 months post-op.		
Case report	2021	Chronic tendinopathy	Patellar	Augmenting patch	1	12	0%	Patient fully returned to all activities without any pain.	None	146
Case report	2021	Chronic tendinopathy	Proximal hamstring	Augmenting patch with injection of platelet-rich plasma	1	12	0%	Patient continued swimming and running pain-free without recurrence with gradual improvement in her pace.	None	146
Case series	2021	Partial-thickness tear	Rotator cuff	Augmenting patch	241	12 (83.5% follow-up)	1.24%	Patient-reported Outcome Measures:	11 revision surgeries due to: 5 shoulder stiffness/adhesive capsulitis; 3 clinically significant bursitis; 3 retear/failure to heal and 1 dislodged graft.	175
								VAS pain score: 5.3 (pre-op) vs 1.1 (post-op) (p < 0.001);		
								SANE score:41.7 (pre-op) vs 86.2 (post-op) (p < 0.001);		
								VR-12 PCS score:35.3 (pre-op) vs 49.2 (post-op) (p < 0.001);		
								ASES pain score: 5.5 (pre-op) vs 1.1 (post-op) (p < 0.001);		
								ASES function score:14.1 (pre-op) vs 26.1 (post-op) (p < 0.001);		
								ASES shoulder score:46.8 (pre-op) vs 88.1 (post-op) (p < 0.001);		
								WORC score: 36.4 (pre-op) vs 83.7 (post-op) (p < 0.001);		
Prospective multicenter	2014-2019	Full-thickness tear	Rotator cuff	Augmenting patch	114	12	16.5%	Patient-reported and Physician-assessed Outcome Measures:	9 reoperation of index shoulder: 7 for symptomatic recurrent or persistent rotator cuff tears; 1 swelling and drainage in the operated shoulder and 1 inflammatory changes and osteopenia in the greater tuberosity region.	176
								CMS scores: 50.4 (pre-op) vs 71.2 (post-op) (MCID = 86.4%);		
								Patient-reported Outcome Measures:		
								ASES shoulder score: 57.3 (pre-op) vs 86.9 (1-year post-op) (MCID = 91.7%);		
								Patient satisfaction: 96.5%		
Prospective multicenter	2021	Full-thickness tear	Rotator cuff	Augmenting patch	191	12	5.7%	Patient-reported Outcome Measures:	22 revision surgeries due to: 11 retear/failure to heal; 3 infection, 3 shoulder stiffness/adhesive capsulitis, 1 clinically significant bursitis, 1 implant displacement, proximal humerus fracture/partial subscapularis tear, and 1 postoperative adhesive capsulitis.	177
								SANE score: 40.0 (pre-op) vs 82.0 (post-op) (p < 0.001);		
								VR-12 PCS score: 33.5 (pre-op) vs 47.3 (post-op) (p < 0.001);		
								ASES shoulder score: 46.2 (pre-op) vs 87.8 (post-op) (p < 0.001);		
								WORC score: 36.2 (pre-op) vs 81.0 (post-op) (p < 0.001)		
Case report	2023	Partial-thickness and full-thickness tear	Rotator cuff	Augmenting patch	1	12	NA	Improved strength but no advance in ROM	1-year postoperatively, patient report dull and aching pain, along with swollen shoulder since surgery. There was significant swelling with debris in the subacromial bursa, containing rice bodies and unhealed portion of the implant. Arthroscopic subacromial debridement was then performed.	178
**TenoMend™ Collagen Tendon Wrap**										
Prospective	2016	Partial-thickness tear	Supraspinatus tendon	Augmenting patch	13	24	0%	Patient-reported and Physician-assessed Outcome Measures:		179
								CMS score improvement: p ≤ 0.01;		
								CMS pain score improvement: p ≤ 0.001.		
								Patient-reported Outcome Measures:	1 excessive swelling;	
								ASES total score improvement: p ≤ 0.001;	1 significant pain.	
								ASES pain score improvement: p ≤ 0.001;		
								MRI:		
								Post-op tear size: 70% no tear; 30% low-grade tear;		
								Patient satisfactory: 92%.		
**VersaWrap®**										
Case report	2022	Acute rupture	Flexor pollicis longus	Augmenting wrap	1	26	0%	No adhesion in the area where graft applied;	None	180
								ROM Outcome Measures:		
								Patient with well-aligned composite fist, intact ROM, and full flexion with extension of the thumb interphalangeal joint.		
								Strength Outcome Measures:		
								Key pinch strength: 11 kg vs 8 kg (contralateral side).		
Retrospective	2022-2024	Sharp lacertation	Extensor or flexor tendons	Augmenting wrap	90	1-19.3	0%	ROM Outcome Measures:	None	181
								Mean active ROM: 88.8%;		
								Mean passive ROM: 94.3%;		
								Patient-reported Outcome Measures:		
								Average QuickDASH score: 30.7;		
								Average VAS pain score: 1.3;		
								Mean percent return of function: 87.7%;		
								92.3% of patients rated good or excellent functional outcomes using Strickland and Glogovac criteria.		
**Composite grafts**										
**BioBrace**										
Case report	2022	Chronic midsubstance tear	Achilles	Augmenting patch	1	6	0%	ROM Outcome Measures (8-week post-op):	None	105
								Plantar flexion: 35°;		
								Dorsiflexion: 25°;		
								Strength Outcome Measures (8-week post-op):		
								Plantar flexion: 4+/5+;		
								Dorsiflexion: 5+/5+;		
								MRI:		
								Tendon defect almost filled with new tissue well-integrated with the underlying tendon in 6 months post-op.		
Case report	2024	Achilles sleeve avulsion	Achilles	Right ankle: Augmenting patch;	1	12	0%	Patient-reported and Physician-assessed Outcome Measures (6-month post-op):	None	150
				Left ankle: Augmenting patch with FHL tendon transfer				AOFAS score: 34 (pre-op) vs 64 (post-op);		
								ROM Outcome Measures (6-month post-op):		
								Dorsiflexion: 13° (right) and 10° (left);		
								Plantarflexion: 55° (right) and 55° (left);		
								Inversion: 35° (right) and 40° (left);		
								Eversion: 15° (right) and 15° (left);		
								Strength Outcome Measures (6-month post-op):		
								Plantar flexion strength: 5/5;		
								MRI (6-month post-op):		
								Tendon appeared hypointense and with smooth margins;		
								Ambulatory ability:		
								Patient was able to perform a double legged heel rise at 6-month post-op;		
								Patient returned to work and normal footwear and denies pain 12-month post-op.		
**TAPESTRY® Biointegrative Implant**										
Prospective	2022	Primary glenohumeral osteoarthritis	Subscapularis tendon	Augmenting patch	5	6	20% partial re-tear	Patient-reported Outcome Measures:	None	182
								ASES score: 33 (3-month post-op) vs 35 (6-month post-op);		
								Ultrasonography:		
								No evidence of graft at 6 months post-op.		
Case report	2024	Calcified insertional Achilles Tendinopathy	Achilles	Augmenting wrap followed by FHL tendon transfer	1	6	0%	Patient-reported Outcome Measures:	Plantar fasciitis was reported at 6-month post-op, treated with steroid injection.	183
								VAS score: 2 (3-month post-op) and 4 (6 month post-op);		
								Patient returned to daily activity with minimal restrictions and reported high satisfaction.		

Abbreviation: AOFAS – American Orthopaedic Foot and Ankle Society, scale: 0-100, 100 = best; ASES – American Shoulder and Elbow Surgeons, scale: 0-100, 100 = best; ATRS – Achilles Tendon Total Rupture Score, scale: 0-100, 100 = best; BM-MSCs – bone marrow derived mesenchymal stem cells; CMS – Constant-Murley Score, scale: 0-100, 100 = best; FHL – flexor hallucis longus; IKDC score – International Knee Documentation Committee score, scale: 0-100%, 100% = best; IS-I – Insall-Salvati Index, normal range: 0.8-1.2; KSS – Knee Society Score, scale: 0-100, 100 = best; Lysholm Knee Scoring scale, scale: 0-100%, 100% = best; MCID – minimal clinically important difference; MCS – mental component summary; MRC – Medical Research Council; MRI – Magnetic Resonance Imaging; NA – not available; OSS – Oxford Shoulder Score, scale: 0-48 or 12-60, 48/60 = best; QuickDASH – Quick Disabilities of the Arm, Shoulder and Hand, scale: 0-100, 0-best; SF-12 – Short Form 12 scores; SF-12 PCS – SF-12 physical component summary, >50 = better-than-average health, <50 = below-average health; SST – Simple Shoulder Test, scale: 0-100, 100-best; SSV – Subjective Shoulder Value, scale: 0-100, 100 = best; ROM – range of motion; SANE: Single Assessment Numeric Evaluation, scale: 0-100, 100 = best; UCLA Shoulder score – University of California, Los Angeles Shoulder score, scale: 0-35, 35 = best; VAS – Visual Analog Scale, scale: 0-10, 0 = ’no pain’; VR-12 PCS – Veterans RAND 12-Item physical components; WORC Index– Western Ontario Rotator Cuff Index, scale: 0 = −100, 100 = best.

Allografts are commonly sourced from dermal matrices [119,120] and have been used to both augment and bridge irreparable tendon tears [154]. These grafts usually trigger minimal inflammation and result in positive clinical outcomes [104,153,154,156,185], with the caveat of occasional triggering of a giant cell reaction that can cause significant pain necessitating revision surgery for debridement [186].

Xenografts from decellularized dermis [121,124] and pericardium [122,123] from porcine, bovine and equine sources are the most widely used grafts for rotator cuff repairs [122,124,126,127,135,187]. They need to be carefully monitored for xenogenic content to avoid triggering foreign body reactions at the repair site that can cause complications and even device failure [134,159,[188], [189], [190]].

These events make synthetic grafts attractive, such as those made of synthetic and biological polymers in the form of fibrous patches [130,[132], [133], [134],^142^,^144^], braided scaffolds [135,191,192], knitted scaffolds [[136], [137], [138], [139], [140], [141]], woven tapes [142,143], sheets [114,115,117,144,145,147,148], hydrogels [116], and composite structures [149,151]. Synthetic grafts are known for their mechanical strength and have been shown to improve clinical outcomes in tendon healing as bridging [[130], [131], [132], [133], [134],^144^] and augmenting grafts [193,194]. However, occasional re-tears of tendons repaired with synthetic grafts have been reported [138,166,167,170]. Additionally, cases of delayed foreign body reactions, between 2 and 14 years postoperatively, have been observed [193,194]. These reactions lead to excessive fibrosis along with the accumulation of inflammatory giant cells around the repaired tendon and have necessitated excision followed by tendon reconstruction [193,194]. These outcomes point to the need for longitudinal follow-up studies to predict the fate of synthetic grafts post-implantation.

Biologically inspired grafts are typically composed of reconstituted type I collagen, which is appealing because this content mimics the composition of tendon tissue and facilitates integration [172,179]. They are primarily used as augmenting grafts and have resulted in tear size reduction and functional score improvement in repaired tendons [146,[171], [172], [173], [174], [175], [176], [177],^179^,^180^]. However, these grafts have limited capacity when it comes to repairing medium to massive-sized tendon tears [[171], [172], [173],^175^,^179^] and can induce fluid accumulation at the implant site which results in swelling [171,173,176,179]. For these reasons, composite grafts consisting of both synthetic and biological materials have been developed to augment tendon repair but relatively limited clinical outcomes are available [105,182].

## 4 Current strategies for tendon scaffold engineering

While commercial grafts can enhance tendon repair and enable patients to return to their daily activities, in many cases the functionality of the repaired tendon may not be completely restored and some pain may persist [154,157].

Given the persistent limitations of available tendon grafts, ongoing research is focused on the development of tissue engineered bridging and augmenting grafts. An ideal tendon graft must exhibit appropriate suture retention strength to mitigate the risk of suture “pull-through” at the interface between the suture and tendon [195]. The scaffold should not contain allogenic or xenogenic materials in order to avoid triggering an aggressive immune response post-implantation [196]. It should also exhibit an optimal degradation rate that aligns with the tissue regeneration process, thereby providing the necessary support required during the early phases of tendon healing [196]. Additionally, it is essential for the scaffold to be customizable for patient- and site-specific applications, easily managed by surgeons, and remain stable during storage to ensure clinical safety and efficacy [12].

In addition to the aforementioned requirements for tendon grafts, specific properties are required for each type of graft. A bridging graft should serve as a scaffold, which is the material that provides the structural support for cell attachment and subsequent tissue development [197], for tendon regeneration. Therefore, it should deliver mechanical strength to maintain structural integrity during tendon regeneration [198]. It should also have optimal porosity and pore size for cell infiltration and matrix formation [39], along with an aligned topography to direct tenogenic differentiation of TSPCs and prevent aberrant differentiation [[199], [200], [201], [202]]. Additionally, it is desirable to recapitulate the hierarchical structure of the tendon to encourage tendon fascicle regeneration and restoration of the sliding motion within tendons [52]. In contrast, augmenting grafts should exhibit appropriate mechanical performance to provide sufficient structural strength at the time of implantation to withstand 20-30% of the load experienced by the repaired tendon, thereby preventing re-tear [187,195,203]. Additionally, they should be flexible, to conform to the shape of the tendon and should exhibit low surface friction with surrounding tissues to facilitate smooth tendon excursion and minimize the risk of adhesion [17]. Through incorporation of bioactive adjuncts, augmenting grafts can also potentially be used to promote tendon regeneration, prevent fibrosis and limit adhesion formation [17,156].

**Fig. 2:**
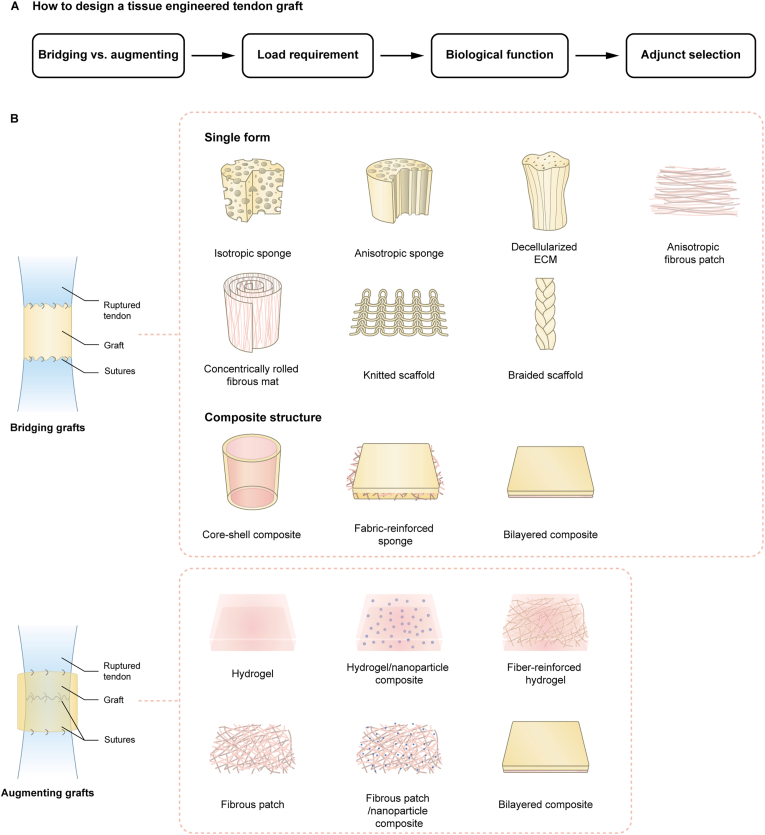
A. How to design a tissue engineered tendon graft; B. Structural composition of prototype bridging and augmenting tendon grafts.

When developing functional tissue engineered tendon grafts, the initial consideration should be the graft type (Fig. 2A). Subsequently, the material and structural design will be determined based on the graft type, load requirements, and the desired biological functions of the graft. Finally, the incorporation of bioactive adjuncts can be considered to fulfil additional biological functions.

### 4.1 Material selection

In tendon tissue engineering, the grafting materials are intended to interact with host cells and subsequently integrate into the repaired tendon *in vivo*. Tendon grafts are typically made from materials broadly categorized as either synthetic or biological as well as a composite of the two. Commonly used synthetic materials include polycaprolactone (PCL) [204], polyglycolic acid (PGA) [205,206], polylactic acid (PLA) [205,206], poly (L-lactic acid) (PLLA) [207,208], poly (lactide-*co*-glycolide) (PLG) [46] and poly (lactic-*co*-glycolic) acid (PLGA) [209]. Biological materials that have been used focus on collagen [35,38,40,208,[210], [211], [212], [213], [214]], silk fibroin [35,210,215], gelatin [207], gelatin methacryloyl (GelMA) [44,99,215,216], alginate [41,217,218], hyaluronic acid [44,180,219] and chitosan [33,201,208]. The advantages and disadvantages of synthetic and biological materials have been extensively discussed elsewhere [19,220]. In the context here, synthetic materials exhibit higher mechanical strengths that are closer to that of tendons [19] with properties that are easy to optimize chemically and reproducibly. However, they often lack biologically functional groups, resulting in less favorable cell attachment and proliferation and, consequently, tissue integration. In contrast, biological materials can be selected on the basis of their content of binding sequences that promote host cell attachment, proliferation, migration, and differentiation [221] but their mechanical performance is often less than that of synthetic materials, and as they are biologically sourced, there can be batch-to-batch variations. Furthermore, the degradation rate of biological grafts is usually faster than that of the deposition of new tissue *in situ*, resulting in the loss of structural integrity before repair is complete [35]. Given their complementary characteristics, combining biological materials with synthetic materials is attractive: it has the potential to produce a synergistic effect that reflects the beneficial characteristics of both polymers [36,41,77,201,202,222,223]. For example, the incorporation of PLA in a hybrid PLA/collagen yarn resulted in a yarn with a tensile strength of 8.7 N, 6.7 times higher than that of the collagen yarn alone [224], while maintaining capacity to support tenocyte proliferation *in vitro* [224].

### 4.2 Structure of tendon grafts

The structure of a tendon graft will influence its effectiveness in facilitating tendon healing and functional regeneration. A suitable configuration ensures that the graft provides adequate mechanical support, prevents re-rupture and allows for natural movement. It can also affect cellular interactions, such as cell infiltration, proliferation, ECM deposition, tissue remodeling [39,199], and the inflammation response [225] (Fig. 2B).

#### 4.2.1 Bridging grafts

Bridging grafts have been designed as either simple single-form structures, such as isotropic and anisotropic sponges [38,46,73,211,212,226], decellularized ECM [37,227], anisotropic fibrous patches [40,201,213], concentrically rolled fibrous mats [204,213], knitted scaffolds [36,210], and braided scaffolds [34,77], or as complex composite structures like core-shell composites [33,202,[204], [205], [206],^211^], fabric-reinforced sponges [35,210,211,[228], [229], [230]] and bilayered composites [231,232] (Table 3).

**Table 3:** Tissue engineered tendon bridging grafts under development. *In vivo* outcomes are compared to untreated injured tendon, unless otherwise specified.

Single-form structure				
Graft structure	Material/Component	Mechanical properties	Outcomes in repaired tendon	Ref
**Isotropic sponge**	Collagen, BMSCs (stimulated with TGF-β1 and cyclic mechanical stretch *in vitro* for 3 days before implantation)	Failure load: 0.48 ± 0.20 N;	•Better macroscopic score •Better histology score •Better Achilles functional index •Improved Young's modulus •Larger area of regenerated collagen fibers •Inferior Young's modulus and histology score compared to normal tendon	212
		Failure strength: 16.98 ± 3.42 kPa;		
		Failure strain: 94.10 ± 18.99 %;		
		Young's modulus: 20.35 ± 8.12 kPa.		
**Anisotropic fibrous patch**	Collagen, periostin	Failure force: 33.31 ± 2.06 N;	•A smooth surface with deposition of compact and aligned ECM, resembling that of a native tendon •Better Achilles functional index •Improved failure stress, failure load and elastic modulus •Surface Young's modulus at nanoscale comparable to native tendon •Large area of deposited tenascin-C and tenomodulin •Enhanced neovascularization •Prevented heterotopic ossification •Inferior failure force, failure stress and elastic modulus compared to normal tendon	40
		Failure stress:10.60 ± 0.65 MPa;		
		Elastic modulus: 11.15 ± 1.08 MPa.		
**Concentrically rolled fibrous mat**	Crosslinked collagen, rBMSCs	Tensile strength: 18.45 ± 0.91 MPa;	•Better histology score •Better Achilles functional index •Higher expression of tendon-related genes, including *Col1a1*, *Bgn*, *Tnc*, *Scx*, *Tnmd* and *Dcn* Higher expression of tendon-related proteins, including SCX and TNMD •Improved failure load, failure stress, failure strain, stiffness and Young's modulus •Inferior histology score than normal tendon •Thickness of the repaired tendons were larger than normal tendons •Expression of tendon-related genes were lower than normal tendons	213
		Strain at failure: 28.33 ± 0.81 %;		
		Young's modulus: 79.19 ± 9.39 MPa.		
**Decellularized ECM**	Decellularized tendon, rBMSCs sheets	Not reported	•Better histology score •Higher density of type I collagen fibers •Smaller cross-sectional area •Improved failure load, ultimate strength, stiffness and Young's modulus •No comparison with normal tendons	232
	Decellularized tendon, TDSCs-derived ECM	Not reported	•Better Achilles functional index •Similar failure strain, Young's modulus, and failure load •Higher M2:M1 macrophage ratio in early stage of healing •Lower concentration of pro-inflammatory cytokines including IL-6 and IL-1β in early stage of healing •Higher concentration of anti-inflammatory cytokine, IL-6 in later stage of healing •Higher failure strain than normal tendon •Comparable Young's modulus and failure load as normal tendon	37
**Composite structure**				
**Bilayered composite**	Two hydrogel layers made from skin secretions of *Andrias davidianus*	Storage modulus:	•Better histology score •Improved collagen organization •Improved tensile strength, Young's modulus, stiffness and failure load •Improved gait performance, including maximum contact area, maximum intensity and mean intensity. •More proliferative (Ki67+) cells •Similar amount of type I collagen but more type III collagen •Inferior gait performance compared to normal tendon •Inferior mechanical properties compared to normal tendon	231
		Inner layer: 1229 ± 411 Pa;		
		Outer layer: 2412 ± 472 Pa;		
		Loss modulus:		
		Inner layer: 420 ± 230 Pa;		
		Outer layer: 476 ± 134 Pa.		
**Core-shell composite**	Shell: PCL mesh	Not reported	•Better macroscopic scores •Better histology scores •Improved Achilles functional index •Improved failure force and Young's modulus, but similar stiffness •Larger thickness •Similar weight •Enhanced expression of *Aspn*, *Tnmd*, *Col1* and *Col3* •Enhanced expression of COL1A1 and TNMD •Higher area percent of collagen 1 •Thicker collagen fibers •Collagen fibrils are thinner than that in normal tendons •Area fraction of type I collagen was smaller than normal tendon	202
	Core: anisotropic GelMa hydrogel + TSPCs			
	Shell: compact chitosan film	Tensile modulus: 13.62 ± 1.69 MPa;	•Improved ultimate stress and tensile modulus •Lower cell density •Better adhesion score •Inferior tensile modulus than normal tendon	33
	Core: porous chitosan sponge	Breaking Strength: 1.36 ± 0.08 MPa.		
	Shell: GelMa hydrogel + soluble tendon ECM extract	Maximum load: ∼60N;	•Improved gait performance including contact area, print width, stride length, and print intensity •Ultimate load and stiffness comparable to intact tendon, no comparison with empty defect group	233
	Core: polyurethane elastomer	Linear stiffness: ∼50 N/mm;		
		Tensile modulus: ∼400 MPa.		

**Abbreviation**: BMSC – bone marrow-derived stem cells; ECM – extracellular matrix; PCL – polycaprolactone; GelMa – gelatin methacryloyl; rBMSC – rabbit bone-marrow-derived stem cells; TDSCs – tendon-derived stem cells; TGF-β1 – transforming growth factor-beta 1; TSPCs – tendon stem/progenitor cells.

**Fig. 3:**
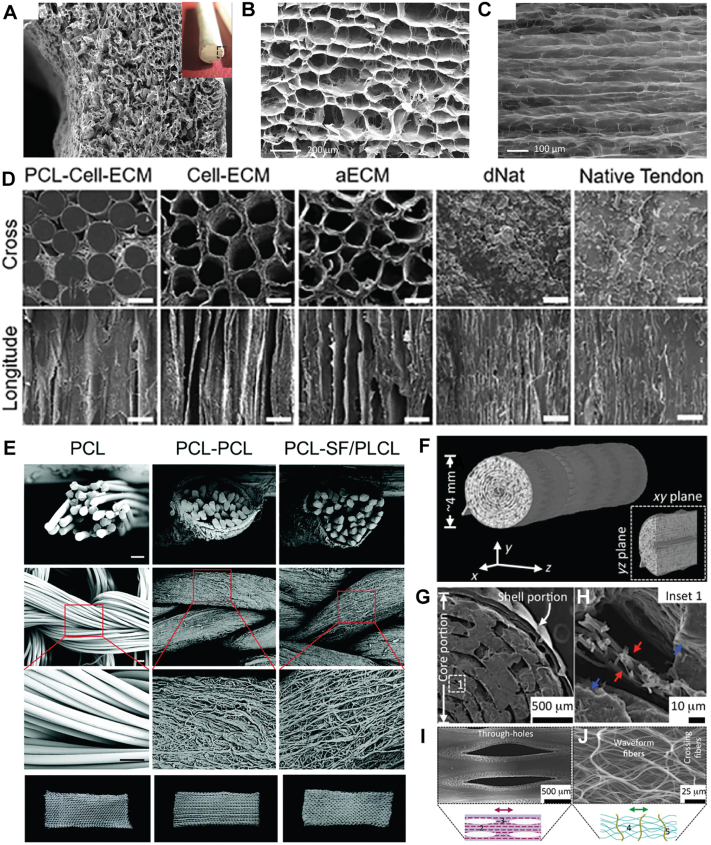
Structures of tendon bridging grafts. **A-C**. Structure of a tubular sponge prepared via unidirectional freezing. Adapted with permission under CC BY license from Ref. [211]. **A**. Micro and macro (insert) structure of the sponge's transverse section. **B**. High-resolution transverse section of the pore microstructure. **C**. Longitudinal view of the tubular sponge. **D**. Micro-CT scans of the transverse and longitudinal cross-sections of a synthetic allogenic decellularized matrix. The gaps between aligned PCL microfibers were filled with dense host tissue in the PCL-Cell-ECM sample. The removal of the PCL template resulted in the formation of a Cell-ECM composite with aligned microchannels. After cell removal, an autologous ECM (aECM) with preserved ECM architecture and spatial orientation was obtained. In contrast, no obvious pore structure was observed across the decellularized native rat tendon (dNat) and native tendon. Scale bar is 100 μm. Adapted with permission from Ref. [39]. **E**. A knitted scaffold made of microfiber/nanofiber core-sheath yarns. The first row shows the SEM images of the PCL microfibers, PCL microfibers-PCL nanofibers (PCL-PCL), and PCL microfiber-silk fibroin/poly (L-lactic acid-co-ε-caprolactone) nanofiber core-sheath yarn (PCL-SF/PLCL). The second and third rows show the SEM images of the knitted scaffolds made from these yarns and the fourth row shows images of the knitted scaffolds. Scale bar is 50 μm. Adapted with permission from Ref. [36]. **F-J**. A core-shell scaffold consisting of a PCL film as the shell portion and a concentrically rolled PCL mesh as the core portion. Adapted with permission under CC BY license from Ref. [204]. **F**. Micro-CT images of a core-shell scaffold exhibiting well-assembled laminae. **G**. SEM images of the cross-sections showing a multilaminar structure, with a single film layer as the shell portion and multiple fiber mesh layers separated by PEO interlayers constituting the core portion. **H**. A close-up image of the core portion. Red and blue arrows within Insert 1 represent the fiber and PEO layers, respectively. **I**. SEM images of the shell portion with elongated through-holes along the stretching direction (double-headed arrow). **J**. SEM images of the core portion with oriented waveform fibers (inset 4) along the stretching direction (double-headed arrow).

Sponges have a highly interconnected, three-dimensional porous network that facilitates cell infiltration and attachment, nutrient diffusion, waste removal, and tissue regeneration [33]. They can be produced through diverse methods such as solvent casting [46], lyophilization [38,230], and freeze-thaw cycles followed by lyophilization [35,46]. The porosity and pore size of the sponge is typically tailored by adjusting the concentration of the pre-polymer solution [35] and utilizing salt infusion/salt leaching [46]. However, an isotropic sponge does not replicate the aligned architecture of a native tendon, and this mismatch can lead to the regeneration of a disorganized ECM [35]. Therefore, sponges with aligned microchannels have been developed using unidirectional freezing [35,73,211,226] that exhibit enhanced mechanical strength and promote the deposition of highly organized ECM consisting of mature and dense collagen fibers, compared to their isotropic counterpart [35] (Fig. 3A–C).

Decellularization of tendon explants generally preserves their structural alignment, biochemical composition and biomechanical properties, which can promote the tissue-specific differentiation of endogenously recruited progenitor cells following implantation into a tendon repair site [37,227,234]. However, there is an increased risk of process-driven reduced porosity and pore size in the tendon ECM, which can restrict cell infiltration, induce persistent inflammation and result in the formation of a mineralized, type II collagen-based tissue [39]. The xenogeneic nature of donor ECM can compound problems by inducing inflammation reactions [159]. To address these issues, a synthetic allogenic decellularized matrix with a desired pore size and porosity has been fabricated [39] by subcutaneously implanting an aligned PCL template in rats for four weeks to obtain a PCL-cell-ECM composite [39,235] and then, removing the PCL with chloroform and the cellular components through repeated freeze-thaw cycles and enzymatic treatments, leaving an ECM scaffold with aligned microchannels [39] (Fig. 3D). Compared to decellularized native rat tendons, this approach gives a significantly larger mean pore size and porosity, easing cell infiltration into the interior of the ECM scaffolds following implantation into a damaged rat tendon [39]. In these studies, although the mechanical strength of the resulting matrix was still inferior to a rat tendon, this approach has potential for the production of decellularized autogenic scaffolds consisting of microchannels with controllable dimensions.

Fibrous bridging grafts usually comprise micro-scale and/or nanoscale fibers, mimicking the collagen fibers in the tendons [67] and are fabricated as anisotropic fibrous patches [40,201,213], fiber bundles [205,206], textile-like scaffolds [34,77], or composites of these structures [205,206]. Subsequently, they are often processed into concentrically rolled fibrous mats before implantation [204,213]. Electrospinning is one of the most useful techniques to produce fibrous scaffolds for tendon applications [236] but conventional electrospinning typically gives randomly aligned fibers, which at least *in vitro* suppress the expression of tendon ECM components in both tenocytes [199] and bone marrow-derived stem cells (BMSCs) [213], induce polarization of macrophages towards a pro-inflammatory phenotype [225] and increase the expression of matrix metalloproteinases (MMPs) in tenocytes in a co-culture with M1 macrophages [199]. In contrast to randomly aligned scaffolds, aligned scaffolds enhance the expression of tendon-related ECM genes such as *Col1a1* [213], *Bgn* [213], *Tnc* [213], *Scx* [213], *Tnmd* [213], *Dcn* [201,213], and *Fmod* [201] in a rat Achilles defect model, resulting in improved repaired tendons with thicker collagen fibrils [201] and increased stiffness [201,213]. As a consequence, research has focused on the alignment of fibers through methods such as mechanical stretching after electrospinning [204,237]. Alternatively, anisotropic fibrous patches consisting of aligned fibers can be produced by electrospinning onto rotating drums, stable jet electrospinning [45,215,222,238], counter-rotating extrusion [213], and dynamic diffusion-based self-assembly [40]. Their mechanical strength can be enhanced by the incorporation of synthetic fibers during the electrospinning process [34,200]. Yarn-like fiber bundles containing oriented microfibers and nanofibers can be produced via electrospinning and electrochemical alignment with a rotating electrode device [224,239]. They can then be processed into textile-resembling scaffolds via braiding [34,77], knitting [36,210], and weaving [224] (Fig. 3E). These processes have the added benefit of significantly enhancing the mechanical strength of the scaffolds [36,224].

It is challenging to simultaneously achieve robust tendon regeneration and adequate mechanical support in the aforementioned single-form structures, and as a result tendon grafts with composite structures with two or more components have been developed (Table 3). They include fabric-reinforced sponges, bilayered composites, and core-shell composites. The mechanical strength of porous sponges has been increased, for example, by embedding knitted scaffolds [35,210,228,229], nonwoven fabric [230], or braided scaffolds [211] within the sponge. Bilayered composites are commonly used as an augmenting tendon graft (*vide infra*).

Core-shell composite structures are increasingly considered for tendon grafts as they integrate the properties of both the core and the shell while also mimicking the hierarchical structure of a tendon. The therapeutic effects of these composite grafts can be tailored by optimizing the structures of the shell and core portions. For example, to prevent peritendinous adhesion formation, the shell layer can be designed as a compact film that provides mechanical support while impeding extrinsic cell infiltration from the surrounding tissue [33] and the core portion can be fabricated as a porous sponge that allows intrinsic cell infiltration from the tendon stumps. Another design utilizes a bioactive hydrogel as the shell portion to encourage cell infiltration and proliferation, while the core portion is made from a synthetic polymeric sponge that provides robust mechanical support and undergoes slow degradation [233]. In other examples, porous mesh shell layers have been used to hold loosely packed cores containing either bundles of fibers [205,206] or concentrically rolled films together [202,204] to encourage cell infiltration to the core (Fig. 3F–J). These structural differences in the shell and core portions have the ability to confer physical cues that guide the regeneration of ECM with distinct organization, mimicking the epitenon and tendon core [204].

Inspired by fascicles in the tendon, researchers have developed multiscale compartmentalized core-shell composites [240]. These scaffolds consist of aligned electrospun PLLA patches that are rolled concentrically to form fiber bundles and then further wrapped in a PLLA nanofibrous sheath [240]. The porous sheath of nanofibers allows for cell growth and migration into the interior of the scaffold *in vitro* [240]. The multiscale hierarchical scaffolds exhibit a similar stiffness to natural tendons but a lower yield stress and failure stress, showing potential for the regeneration of tendon tissues with multiple fascicles *in vivo* [240].

#### 4.2.2 Augmenting grafts

Augmenting grafts are typically flexible patches that complement the shape of a tendon. They are usually fabricated as hydrogels [99,214,241], hydrogel/nanoparticle composite [242,243], fiber-reinforced hydrogels [244], fibrous patches [45,215,222,238,245], fibrous patch/nanoparticle composite [246], or bilayered composites [41,44,47,100,102,209,247] (Table 4) and are often used as delivery systems for the release of bioactive adjuncts to accelerate tendon healing and prevent adhesion formation.

**Table 4:** Augmenting tendon grafts under development.

Structure	Components	Reference
**Hydrogel**	HA-chitosan-PNIPAM	241
	Collagen, TNF-α primed ASC-derived EVs	214
	GelMa, MSC-EVs	99
**Hydrogel/nanoparticle composite**	**Nanoparticle**: COX-1 and COX-2 miRNAs, PLGA	242
	**Hydrogel**: HA-PEG	
	**Nanoparticle**: COX-1 and COX-2 siRNAs, PLGA	243
	**Hydrogel**: HA-PEG	
**Fiber-reinforced hydrogel**	**Fiber**: Collagen	244
	**Hydrogel**: Alginate	
**Fibrous patch**	SF/GelMa, MSCs	215
	PLGA, Cell membranes of CD68^+^ inflammatory macrophages	45
	Celecoxib, PELA	238
**Fibrous patch/nanoparticle composite**	**Nanoparticle**: Ibuprofen, MMS	246
	**Fibrous patch**: PLLA	
**Fibrous patch with core-shell nanofibers**	**Shell**: PLLA	245
	**Core**: mitomycin-C, HA hydrosols	
	**Shell**: PELA	222
	**Core**: miR-29a embedded lipid nanoparticles mixed with HA	
**Bilayered composite**	**Outer layer**: DegraPol® fibrous patch	247
	**Tendon-facing layer**: PDGF-BB, DegraPol® fibrous patch	
	**Outer layer**: Ibuprofen-containing PLGA fibrous patch	209
	**Tendon-facing layer**: bFGF-containing PEG-PLV hydrogel	
	**Outer layer**: alginate-containing PAAM hydrogel	41
	**Tendon-facing layer**: triamcinolone acetonide-containing chitosan hydrogel	
	**Outer layer**: TGF-β1 siRNAs-embedded G5-GBA nanoparticles loaded into a carboxymethyl chitosan hydrogel	47
	**Tendon-facing layer**: PCL fibrous patch	
	**Outer layer**: Smad3 siRNA-embedded G5-GBA nanoparticles loaded into GelMa microspheres then mixed within an HA hydrogel	44
	**Tendon-facing layer**: PCL fibrous patch	
	**Outer layer**: CRISPR-Cas13 RNP-loaded bPEI-PBA/bPEI-Man nanoclusters embedded in an HA-PBA/PVA hydrogel	102
	**Tendon-facing layer**: PCL fibrous patch	
	**Outer layer**: PCL fibrous patch	100
	**Tendon-facing layer**: HA-ADH and PA/Fe hydrogel	

**Abbreviation**: ASC – adipose-derived stem cell; bFGF – basic fibroblast growth factor; bPEI-PBA – 4-(bromomethyl) phenylboronic acid modified branched polyethyleneimine; bPEI-Man – 4-isothiocyanatophenyl α-D-mannopyranoside-modified bPEI; COX – cyclooxygenase; CRISPR – clustered regularly interspaced short palindromic repeats; EV – extracellular vesicle; Fe – iron ions; HA – hyaluronic acid; HA-ADH – hyaluronic acid-adipic acid dihydrazide; G5-GBA – guanidinobenzoic acid (GBA) modified generation 5 polyamidoamine; GelMa – gelatin methacryloyl; HA-PBA – 3-aminophenylboric acid-modified hyaluronic acid; MMS – mesoporous silica nanoparticles; MSC-EV – mesenchymal stem cell-derived extracellular vesicles; PA – protocatechuic aldehyde; PAAM – polyacrylamide; PCL – polycaprolactone; PDGF-BB – platelet-derived growth factor BB; PEG – poly(ethylene glycol); PEG-PLV – methoxy poly(ethylene glycol)-block-poly(L-valine); PELA – polylactic acid-polyethylene glycol copolymer; PNIPAM – poly(N-isopropylacrylamide); PLGA – poly(lactic-co-glycolic) acid; PVA – polyvinyl alcohol; RNP – ribonucleoprotein complex; SPP1 – secreted phosphoprotein 1; TGF-β1 – transforming growth factor–beta 1; TNF-α – tumor necrosis factor-alpha.

**Note**: Bioactive adjuncts that are incorporated within the grafts are highlighted with underlining.

The hydrogels that are employed are water-swollen polymeric materials that maintain a distinct three-dimensional structure after implantation [248,249]. They can be fabricated using bioprinting [202], *in situ* polymerization [99,231], microfluidic construction techniques [44], mould casting [41,209,244], and the self-assembly of peptides [250]. The elastic and soft nature of hydrogels is appealing because they are mechanically gentle to neighboring tissues [251], however, under the compression forces that occur between the tendon and surrounding tissue, implanted conventional hydrogels can be broken down into smaller pieces that recruit pro-inflammatory macrophages [44,173]. To address this limitation, a range of self-healing and deformable hydrogels has been developed with dynamic covalent bonds that break and reform when the hydrogels undergo significant shear or compression forces *in situ* [44,100]. Consequently, these hydrogels can maintain their structural integrity. Another limitation of conventional hydrogels is the requirement for them to be sutured in place, which necessitates a relatively large surgical incision that is complicated by the increased risks of complexity and infection [117]. Such challenges can be reduced by using an injectable hydrogel, which is easier to deliver and time-saving, and thus potentially cost-effective. Injectable hydrogels also allow for more flexible application than does a rigid graft, for the treatment of irregularly shaped defects in tendon surgery [241]. An appealing form of injectable hydrogel uses thermo-responsive polymers that undergo a reversible sol-gel phase transition [241] - they are soluble when the temperature is below their lower critical solution temperature and convert to a gel state above this temperature. Post-injection, at physiological temperatures, the hydrogel solution gels to form a rigid material that can act as a barrier to limit cell penetration and fibroblast proliferation, thereby alleviating peritendinous adhesions in a rabbit deep flexor tendon model [241].

Fibrous patches benefit from their flexibility, which facilitates their use as a material that can be wrapped around a damaged tendon to augment tendon healing [45,215,238]. Additionally, bioactive adjuncts can be incorporated into the structure during fabrication and subsequently released at the injury site to accelerate tendon healing [45,222,238,245].

Augmenting grafts are frequently utilized as bilayered composites, typically integrating hydrogels and fibrous patches to achieve synergistic effects [44,47,100,102,209]. For example, the hydrogel may be used as a tendon-facing inner layer that releases bioactive adjuncts to stimulate intrinsic healing, while the fibrous patch as an outer layer provides a physical barrier that impedes the infiltration of extrinsic cells and thereby reduces peritendinous adhesions [100]. Alternatively, when a hydrogel is placed at the outer layer to supply anti-adhesion agents, the tendon-facing fibrous mesh can be used to restrict the access of these agents to the injured tendon through the use of nanoscale porosity [44,47,102]. This has been demonstrated using a composite that comprises a PCL fibrous patch inner layer with a hydrogel containing TGF-β1 siRNA embedded nanoparticles on the outer layer [47], where the PCL patch blocks nanoparticle access to the tendon but allows them to disperse in the peritendinous region. This inhibits both fibroblast proliferation and collagen deposition in that region which prevents adhesion formation without impeding intrinsic healing at the tendon core [47].

### 4.3 Bioactive adjuncts

The use of bioactive adjuncts is the most researched area in tendon tissue engineering. In addition to the physical cues and mechanical support conferred by a tissue engineered tendon graft, the incorporation of biomolecules further enhances tendon healing and regeneration through the judicious use of biochemical cues. Bioactive adjuncts are molecules that interact with cells and influence their action. Examples of bioactive adjuncts that have been assessed for their potential in tendon regeneration include growth factors, chemokines, ECM proteins, extracellular vesicles (EV), non-coding RNAs including both small interfering RNA (siRNA) and microRNA (miRNA), and anti-inflammatory drugs. The benefits and challenges associated with each type of these bioactive adjuncts are summarized in Table 5. The following discussion will focus on the *in vivo* biological functions of specific adjuncts and their incorporation within a tendon graft.

**Table 5:** Benefits and challenges of different types of bioactive adjuncts.

Types	Examples	Benefits	Challenges	Reference
Growth factor	SDF-1α, PDGF-BB, bFGF, IGF-1, GDF-5, BMP-12, TGF- β1, TGF- β3	−Regulation of wide range of cellular activities −Therapeutic potential in various applications	−Short half-life, retention period and stability −Possibility of side effects at a high local concentration −Lack of cost-effective production method	[252]
EV	TDSC-EVs, BMSC-EV,	−Decrease in cell apoptosis and tissue inflammation −Increase in cell proliferation, angiogenesis and lineage specific differentiation	−Short half-life, retention period and stability −Lack of standardized guidelines on dosage measurement −Difficulty in extraction and isolation −Complex preservation techniques −Low batch-to-batch reproducibility −Limited knowledge on EV heterogeneity −Poorly understood *in vivo* pharmacokinetis or pharmacodynamcs	[[253], [254], [255]]
Non-coding RNAs	siRNA, miRNA	−Targeting of previously undruggable genetic components −Short production time −Prolonged effect through encapsulation −Therapeutic potential for rare diseases −Minimal risk of genotoxicity	−Challenging to reach target organs and achieve efficient cellular uptake	[256,257]
NSAIDs	Ibuprofen, Celecoxib	−Alleviation of pain and inflammation	−Elevated risk of adverse events −Role in tendon healing unclear	[[258], [259], [260]]

**Abbreviation**: bFGF – basic fibroblast growth factor; BMP-12 – bone morphogenic protein 12; BMSC – bone marrow derived stem cell; EV – extracellular vesicle; GDF-5 – growth/differentiation factor 5; IGF-1 – Insulin-like growth factor 1; PDGF-BB – platelet-derived growth factor-BB; SDF-1α – stromal cell-derived factor-1 alpha; TDSC – tendon-derived stem cell; TGF-β1 – transforming growth factor beta 1; and TGF-β3 – transforming growth factor beta 3.

#### 4.3.1 Biological functions of bioactive adjuncts

##### 4.3.1.1 Endogenous tendon cell recruitment and proliferation

Recruiting tenocytes and TSPCs to a tendon injury site is the crucial first step in regenerating a functional tendon matrix [55,61]. Bioactive adjuncts that are incorporated into tendon grafts and encourage endogenous tendon cell recruitment and proliferation at the injury site in *in vivo* studies include stromal SDF-1α [38,229], periostin [40], extracellular vesicles collected from both BMSCs [261] and TDSCs [227] as well as TPSC-derived ECM [37]. *In vitro*, the proliferation of tendon cells is improved using molecules such as platelet derived growth factor – BB (PDGF-BB) [73], bFGF [34,73,200], insulin-like growth factor-1 (IGF-1) [73], SDF-1α [73], tropoelastin [262] and TDSC-EVs [227]. These biomolecules are incorporated either individually or in mixtures [37,39,40,227,231,261,263,264] to synergistically promote tendon healing [73,226]. For example, the delivery of IGF-1 alone to tenocytes cultured on an aligned collagen-GAG scaffold encourages proliferation of tenocytes but downregulates several key tenogenic genes. In contrast, delivery of growth/differentiation factor 5 (GDF-5) alone has little impact on tenocyte chemotaxis, proliferation, or collagen synthesis but promotes a pro-tenocyte phenotype. Beneficially, the combined delivery of IGF-1 and GDF-5 drives both tenocyte proliferation and phenotype maintenance *in vitro* [73].

##### 4.3.1.2 Tendon ECM synthesis

During repair, synthesizing an ECM with a composition and structure that resembles the pre-injured tendon ECM helps to restore the mechanical and biological function of the native tendon [25]. Bioactive adjuncts have been shown to upregulate the gene expression and/or deposition of tendon-specific ECM components *in vitro* and/or *in vivo.* Typically targeted tendon ECM components include type I collagen, tenomodulin, scleraxis, decorin, tenascin C and COMP. Biomolecules that have been incorporated in augmenting grafts to promote ECM synthesis are listed in Table 6. Their presence contributes to a denser fibrous structure [42] with enhanced stiffness and ultimate tensile strength [[270], [271], [272], [273], [274]].

**Table 6:** Bioactive adjuncts that can modulate cell gene expression and/or increase protein deposition of ECM components typically found in tendons.

Targeted tendon ECM Gene/Protein	Function of Protein	Bioactive molecules shown to upregulate gene expression	Bioactive molecules shown to increase protein deposition
**COL1A1/Type I collagen**	−Major component of tendon ECM [49,64] −Provides mechanical strength [52,[64], [65], [66], [67], [68]]	bFGF [77],	bFGF [77,209],
		TGF- β1 [212],	TGF- β1 [212],
		TGF- β3 [265,266],	TGF- β3 [[265], [266], [267]],
		BMP-12 [268],	SDF-1α [38]
		IGF-1 [269],	L-Arginine-HA [42],
		TDSC-derived ECM [37],	Periostin [40],
		IFN-γ-primed MSC-EVs [214]	BMSC-EVs [261],
			IFN-γ-primed MSC-EVs [214], miR-29a [222],
			Autologous decellularized ECM [39]
**TNMD/Tenomodulin**	−Essential for tendon maturation [80] −Regulates cell migration, proliferation, and stem cell senescence during early tendon healing [81] −Regulates the expression of key tendon transcription factors, ECM genes, and proteins [81]	TGF- β1 [212],	TGF- β1 [212],
		BMP-12 [268],	Periostin [40],
		GDF-5 [269],	BMSC-EVs [261],
		Tendon-derived ECM [264],	TDSC-EVs [227]
		TDSC-derived ECM [37]	
**SCX/Scleraxis**	−Unique transcription factor in tendons [90] that regulates the expression of tenomodulin [80], and directs the formation of an organized type I collagen-rich ECM [89]	TGF- β1 [212],	TGF- β1 [212],
		BMP-12 [75,268],	TGF- β3 [265,266]
		GDF-5 [269],	BMSC-EVs [261]
		TGF- β3 [265,266]	TDSC-EVs [227]
		Tendon-derived ECM [264]	
**DCN/Decorin**	−Controls the diameter of collagen fibrils [64,72] −May promote collagen fibrillar slippage and improve lateral interactions during tensile deformation, thus maximizing the load before failure occurs [64,72]	PDGF-BB [74]	
**TNC/Tenascin-C**	−Important in collagen fibril organization [82] −May contribute to tissue elasticity [67]	bFGF [77],	bFGF [77],
		TGF-β1 [212],	TGF-β1 [212],
		BMP-12 [268],	TGF-β3 [265,266]
		TGF-β3 [265,266]	SDF-1α [38],
		Tendon-derived ECM [264],	Periostin [40],
		TDSC-derived ECM [37]	Tropoelastin [76]
**COMP/Cartilage oligomeric matrix protein**	−Most abundant glycoprotein in tendons [67] −Important during tendon development [67,79]	TGF- β3 [265,266]	TGF- β3 [265,266]

**Abbreviation**: bFGF – basic fibroblast growth factor; BMP-12 – bone morphogenic protein 12; BMSC – bone marrow-derived stem cells; COMP – cartilage oligomeric matrix protein; EV – extracellular vesicle; GDF-5 – growth/differentiation factor 5; HA – hyaluronic acid; HUMSC-Exo – human umbilical cord mesenchymal stem cell-derived exosomes; IGF-1 – Insulin-like growth factor 1; PDGF-BB – platelet-derived growth factor-BB; SDF-1α – stromal cell-derived factor-1 alpha; TDSC – tendon-derived stem cell; TGF-β1 – transforming growth factor beta 1; and TGF-β3 – transforming growth factor beta 3.

The utilization of microRNA-29a (miR-29a) has also been investigated in tendon healing, as it attractively downregulates collagen III synthesis with no effect on type I collagen expression by tenocytes under normal physiological conditions [222,275,276]. In an *in vitro* pro-inflammatory environment, a polylactic acid-polyethylene glycol copolymer (PELA) augmenting graft containing miR-29a based nanoparticle increases the deposition of collagen I while reducing both collagen III and alpha smooth muscle actin (αSMA) expression by tenocytes [222]. Application of this construct to augment the healing of suture-repaired rat Achilles tendons results in the regeneration of tendons with enhanced Achilles functional indexes and increased mechanical strength [222].

##### 4.3.1.3 Immunomodulation to promote healing

Transient inflammation is a key component of scarless neonatal tendon regeneration, where macrophages are required to facilitate tenocyte proliferation and restore the mechanical properties of the injured tendon [95]. However, persistent inflammation can impede regeneration and functional recovery and lead to pathologic tissue fibrosis [277,278]. To address this, recent strategies have been focused on reducing M1 macrophage polarization, inducing M2 macrophage polarization [37,99] and modulating cyclooxygenase (COX) activity in the early stages of tendon healing [209,242,243].

A hyaluronic acid hydrogel containing protocatechuic aldehyde, a naturally occurring molecule extracted from the root of the Chinese herb *Salvia miltiorrhiza*, modulates the inflammatory microenvironment in the injured rat Achilles tendon by decreasing the level of proinflammatory cytokines, including TNF-α and IL-6, while increasing anti-inflammatory cytokines, especially IL-4 and IL-10^100^. This corresponded to a reduction in M1 polarization as a result of inhibited NF-*κ*B p65 phosphorylation. With the transition from a pro-inflammatory to an anti-inflammatory microenvironment, the repaired tendons show reduced peritendinous adhesion and improved functionality.

By delivering bioactive glass-elicited MSC-EVs [99] and TDSC-derived ECM [37] to the injury site using hydrogels and decellularized tendons, respectively, macrophages can be polarized to an M2 phenotype. EVs, secreted by bioactive glass-stimulated MSCs, promote early M1-to-M2 phenotypic transition via therapeutic RNAs, miR-125a-5p and miR199b-3p [99], and macrophage-mediated angiogenesis in the early stages of tendon regeneration, resulting in a repaired tendon with mechanical properties comparable to those of a healthy tendon [99]. TDSC-derived ECM also promotes early macrophage polarization to an M2 phenotype, possibly due to the enrichment of anti-inflammatory cytokine IL-4 [37], leading to an enhanced Achilles functional index and improved mechanical strength in the repaired rat Achilles tendon. Despite a lack of clarity on the precise mechanism (including a correlated signaling pathway), these findings highlight the importance of managing the inflammatory microenvironment and macrophage behavior for optimizing tendon regeneration.

COX-1 and COX-2 catalyze the synthesis of proinflammatory prostaglandin E_2_ (PGE_2_) and are upregulated in injured tendon tissue [242,279]. Elevated levels of PGE_2_ can reduce tenocyte contraction *in vitro* [280] and injection of exogenous PGE_2_ leads to degenerative changes within the tendon [281], suggesting a reduction of PGE_2_ in the wound site may enhance tendon healing [280].

Delivering non-steroid anti-inflammatory drugs (NSAIDs) such as ibuprofen, and nucleic acids that suppress both COX-1 and COX-2, with an augmenting graft promotes macrophage polarization towards an M2 phenotype [243] and reduce pro-inflammatory cytokines [209] in the rat Achilles tendon.

In addition to their anti-inflammatory effects, COX inhibitors affect the mechanical strength of the repaired tendons, but variations in the healing outcomes have been reported. Even though delivered ibuprofen inhibits both COX-1 and COX-2, it does not influence the mechanical strength of the repaired tendon at 3 weeks post-surgery [209], yet silencing the expression of both COX-1 and COX-2 using siRNAs or miRNAs enhances the ultimate strength of healed rat Achilles tendons at 3 weeks [243] and chicken flexor tendons at 6 weeks post-surgery [242]. Conversely, a fibrous patch delivering celecoxib that selectively inhibits COX-2 reduces the tensile strength of the repaired tendon by downregulating fibroblast proliferation and collagen synthesis 6 weeks post-surgery [238]. These discrepancies highlight the complexity of using NSAIDs and RNAs to enhance tendon healing, however, the underlying mechanisms were not investigated in these studies.

Similar results were observed in studies on NSAIDs administered by ingestion and injection [238,258,259,[282], [283], [284]], suggesting that the discrepancies could result from a potential inhibitory role of the COX-1-dependent signalling pathway in tendon healing [284,285]. In addition, NSAIDs may affect tendon healing by mechanisms that are unrelated to inflammatory cells [258,259,286,287]. Furthermore, the timing and duration of COX inhibition should be considered: NSAIDs have significantly reduced the mechanical strength of healed tendons when administered during the early inflammatory phase (first 5 days after injury), but have no effect if administered during the remodeling phase (after 6 days post-injury) [288]. These factors should be considered when designing a graft to inhibit COX activity during tendon healing.

##### 4.3.1.4 Reduction of scar/adhesion formation

Fibrotic healing, a common characteristic of tendon repair in adult tendons [27], is marked by the formation of scar tissue consisting of excessive ECM that can be disorganized, with suboptimal biomechanical strength [25]. This problem is compounded in peritendinous adhesions by the attachment of the scar to surrounding tissue and/or the tendon sheath [25]. The two main cellular mediators of fibrosis are myofibroblasts and macrophages [289]. Myofibroblasts are derived from the resident tenocytes and fibroblasts in the latter stage of tendon healing [278,290]. Persistent myofibroblast activity promotes fibrotic healing due to sustained ECM deposition [278,290,291] and the fibroblast-myofibroblast transition can be stimulated by macrophages [289]. Myofibroblast activity in healing tendons can be inhibited by reducing the level of profibrotic transforming growth factor-beta 1 (TGF-β1), inducing fibroblast apoptosis, and limiting profibrotic macrophage activity (Table 7).

**Table 7:** Bioactive adjuncts that reduce fibrotic scar/adhesion formation during tendon healing.

Bioactive adjuncts	Target	*In vivo* outcomes	Reference
siRNA	TGF-β1	•Reduced peritendinous adhesions •No effect on the failure load of the repaired rat Achilles tendon	47
siRNA	Smad3	•Reduced deposition of Smad3, type I collagen and type III collagen in adhesion tissues •Reduced peritendinous adhesions •No effect on maximal strength and stiffness of the repaired rat Achilles tendon	44
miRNA mimics	miR-29a	•Reduced the formation of α-SMA and alleviated peritendinous adhesions •Reduced inflammatory response at the repair site, with fewer infiltrating macrophages and neutrophils and less NF-κB expression •Accelerated tendon healing with higher Achilles functional indexes and failure loads in the repaired rat Achilles tendon	222
Mitomycin-C	Apoptotic gene Bcl-2 and Bax	•Reduced deposition of type I collagen, type III collagen, and α-SMA in the adhesion tissue •Prevented peritendinous adhesions in repaired rabbit FDP and rat Achilles tendon •Reduced work of flexion of the repaired rabbit FDP tendon	245
Ibuprofen	COX-1 and COX-2	•Reduced proinflammatory cytokines including IL-1β, IL-6 and TNF-α •Reduced peritendinous adhesions •No effects on mechanical properties of the repaired tendon	209
miRNA	COX-1	•Reduced peritendinous adhesions •Improved gliding function in repaired flexor tendon •Enhanced ultimate strength of repaired chicken flexor tendon	242
	COX-2		
Celecoxib	COX-2	•Reduced the synthesis of type I and III collagen •Reduced peritendinous adhesions •Improved histological healing score of repaired tissue •Reduced maximal tensile strength of the repaired tendon •Reduced the ratio of work of flexion between the injured and uninjured finger	238
CRISPR-Cas13	SSP1	•Decreased synthesis of type I and III collagen and α-SMA •Reduced peritendinous adhesions	102
Macrophage cell membranes	Pro-inflammatory cytokines	•Reduced the expression of IL-1β and TNF-α in the peritendinous region •Reduced the infiltration of CD68^+^ macrophages and LY6G^+^ neutrophils •Decreased apoptosis of tendon cells at the two ends of the ruptured tendon •Promoted tenocyte proliferation at the early stages of healing •Alleviated the irritation and inflammation of the hind paw after tendon injury •Enhanced the biomechanical strength of the repaired tendon, including stiffness, failure load, and energy •Reduced scar formation	45

**Abbreviation**: ASC – adipose-derived stem cell; α-SMA - alpha smooth muscle actin; COX – cyclooxygenase; CRISPR – clustered regularly interspaced short palindromic repeats; Cas13 – CRISPR-associated 13; ECM – extracellular matrix; FDP – flexor digitorum profundus; IL-1β – interleukin-1 beta; IL-6 – interleukin 6; miRNA – microRNA; NF-κB – nuclear factor-kappa B; siRNA – small interfering RNA; SSP1 – secreted phosphoprotein 1; TGF-β1 – transforming growth factor-beta 1; TNF-α – tumor necrosis factor alpha.

In the remodeling phase of tendon healing, the activated TGF-β1/Smad3 pathway contributes to excessive collagen production [23,292,293], and promotes tenocyte contraction [280] and the recruitment of mesenchymal stromal cells, which subsequently give rise to myofibroblasts [294]. In healing tendons this pathway has been suppressed through the delivery of antisense oligonucleotides [295], siRNAs and miRNAs that focus on reducing *TGFB1* [47,296] and *SMAD3* expression [44,297]. This in turn lowers the expression of type I and III collagen, and thereby delivers a reduction in peritendinous adhesion [44,47,296,297] which is desirable but not ideal, as the mechanical strength of the repaired tendon is often significantly reduced [44,296,297]. It has been postulated that the suppression of this pathway from the onset of tendon healing reduces the recruitment of endogenous tenocytes [56] and diminishes the initial collagen deposition required to re-establish tissue continuity [23,292]. Smad3 activity is implicated in scar and adhesion formation when it is expressed within the tissues of the surrounding tendon sheath, yet promotes tendon healing when expressed within the tendon itself [298], indicating that the effect of the TGF-β1/Smad3 signaling pathway varies depending on the phase and location of tendon healing. Therefore, grafts designed for site-specific delivery of TGF-β1 or Smad3 silencing molecules at a later stage of tendon healing could be a promising strategy to improve tendon repair while reducing adhesion formation.

The activation of fibroblasts into myofibroblasts can be impeded by inducing their apoptosis [245]. For example, a low dose of mitomycin-C, an apoptosis-inducing drug that inhibits fibroblast survival, incorporated into an electrospun PLLA graft for augmentation repair significantly reduces peritendinous adhesions in both rabbit and rat tendon injury models by simultaneously stimulating the apoptosis of exogenous fibroblasts and reducing the synthesis of adhesion-associated collagen and α-SMA [245]. The mechanical strength of this repaired tendon approximates that of tendons repaired by suturing, indicating that low-dose mitomycin-C does not have a detrimental effect on intrinsic tendon healing, and suggests that its delivery with other bioactive molecules is a promising strategy to prevent peritendinous adhesions while promoting intrinsic healing.

SPP1^+^ macrophages are a pro-fibrotic subtype that emerges during the inflammatory stage [101,102]. Reducing the expression of SPP1 in these macrophages can disrupt the SPP1-induced CD44/AKT positive feedback loop in the crosstalk between macrophages and fibroblasts, thereby lowering the expression of α-SMA in fibroblasts during co-culture to subsequently inhibit myofibroblast differentiation [102]. A hydrogel containing CRISPR-associated 13 ribonucleoprotein complex applied to a rat Achilles tendon directly disrupted the expression of SPP1 in macrophages at the post-transcriptional level [102]. This strategy reduces the deposition of type I and type III collagen in peritendinous tissue, which helps prevent adhesions [102].

#### 4.3.2 Delivery of bioactive adjuncts via tendon grafts

While bioactive adjuncts can be injected directly into the injured tendon, their short half-life and poor stability *in vivo* leads to a rapid loss of activity and, there is therefore a demand for frequent dosing [299,300].

The use of grafts as the delivery system for these biomolecules can protect them from rapid degradation and allow for a sustained release profile. Bioactive adjuncts are incorporated into the grafts by either embedding them within the graft material during fabrication or immobilizing them onto the graft surface following fabrication [300,301]. Direct incorporation into fiber-based grafts has been achieved in electrospinning [209,238], co-electrospinning [77], emulsion electrospinning [42,245,247] and co-axial electrospinning [222,247]. Emulsion and co-axial electrospinning generates core-shell fibers that facilitate tailoring of the drug release profile through adjustment of the core and shell composition [42,245,247,302]. Core-shell fibers can also be loaded with two distinct bioactive adjuncts, which may be released at distinct stages of tendon healing to maximize their effectiveness [42,245,247,302].

For incorporation within hydrogels, stable bioactive adjuncts are mixed with the prepolymer solution before the network is physically [209,229] or covalently [41,99,233,242] crosslinked. In contrast, unstable molecules like nucleic acids are often incorporated into nanoparticles [44,242,243] or nanoclusters [102], prior to embedding within the hydrogel [44,222,242]. Incorporated bioactive adjuncts are subsequently released through diffusion and/or following degradation of the hydrogel matrix by sustained delivery [41,99,242]. Hydrogel networks can also be chemically modified to degrade in response to specific environmental cues in injured tendons, such as after exposure to metalloproteinase-2 (MMP-2) [44,47,303] and reactive oxygen species (ROS) [102]. For example, an MMP-2-degradable hydrogel-based augmenting graft that is made by crosslinking alkyl glycidyl ether-modified carboxymethyl chitosan with MMP-2 substrate peptides can be used to deliver nucleic acid-containing nanoparticles [47].

Bioactive adjuncts are immobilized on graft surfaces through physisorption [40,304] or by the use of linker molecules [34,200,226,305]. Physisorption in its simplest form occurs with submersion of the graft in a solution containing the biomolecule to allow for non-specific binding [40,301,304]. However, this attachment process can be readily reversed and therefore usually leads to an initial burst release of bioactive molecules after implantation [40,304].

Surface immobilization through linker molecules is achieved through charge, hydrophobicity, hydrogen bonding, or van der Waals interactions [306]. Heparin has been extensively utilized as a biochemical linker for the delivery of basic fibroblast growth factor (bFGF) [34,200,305], transforming growth factor-beta 2 (TGF-β2) [226] and growth/differentiation factor 5 (GDF5) [226], from tendon grafts. Adapting methods used for bioactive molecules, heparin can be incorporated into the graft by direct incorporation [200], physisorption [223,304,307] or covalent immobilization [34,226]. Alternatively, linker molecules are integrated into hybrid recombinant proteins [38] as seen for example through the addition of a collagen binding domain (CBD) sequence to the C-terminus of the chemokine stromal cell-derived factor 1α (SDF-1α) and its expression in a bacterial system [38]. This incorporation of CBD enables the fusion protein to be bound to a collagen graft non-covalently, allowing for sustained release of the chemokine both *in vitro* and *in vivo* [38].

## 5 Animal models for evaluating engineered grafts

Before their translation to clinical use, the safety and efficacy of engineered tendon grafts are classically evaluated using animal tendon defect models [[308], [309], [310]]. These animal models utilize replicated tendon injuries with a controlled assessment of treatment outcomes [[308], [309], [310]].

### 5.1 Choice of species

The choice of animal model is influenced by the intended repair site and injury type [310]. The most frequently studied models involve injuries to the Achilles tendon, flexor tendon and rotator cuff due to their high prevalence in human tendon injuries [3], which has led to the development of specific animal models to recapitulate this tendon damage [309,310]. For example, the Achilles tendons of rats, rabbits, and dogs, as well as the superficial digital flexor tendon of horses are models for the human Achilles tendon; the flexor tendons of mice [88], rats, dogs and turkeys for the human flexor tendons [311], and the rotator cuffs of rats and sheep for the human rotator cuff [310].

Prior to conducting animal studies, ethical considerations ensure that animal welfare and the relevance of the study to human tendon injury and repair are prioritized. Feasibility assessments are wide-ranging, and take into account factors such as budget, availability of resources/equipment, surgeon capabilities, outcome measures, and study duration [310]. Small animals like mice, rats, and rabbits are used to investigate biological mechanisms that govern tendon development, growth, healing, repair, and aging [27,95]. The ability to modify their genome, particularly in murine models, allows for probing of the roles of specific genes in tendon development, repair, and regeneration [81,298,312,313]. Small animal studies are well-suited for investigating the efficacy of tissue engineered grafts as proof-of-concept studies before moving to more costly, time-consuming, and resource-intensive large animal studies [308,310]. Large animals like the sheep and horse are used as clinically relevant models because of their comparable dimensions and biomechanics to humans which allows investigations into the outcomes of surgical and rehabilitation techniques; and the use of devices, and tissue engineered grafts to treat tendons that are similar in size, anatomy, function, and metabolic activity to those found in humans, and with a comparable rate of healing [310,314].

### 5.2 Injury model

The induced injury should consider the therapeutic application of the prototype tendon grafts. For a bridging graft, transection and partial transection models are frequently used [36,37,40,204] to mimic the complete [2,17] and partial [315] tendon ruptures seen in human injuries, respectively. For augmenting grafts that are designed to wrap around the injury site, tendon rupture and crush injury models are utilized [44,99,215]. For example, tendon injury in animal models can be induced chemically with collagenase to mimic chronic tendinopathy [316] or mechanically through overloading to model overuse injury [317]. Table 8 summarizes commonly used animal models for evaluating the efficacy of tendon grafts, key surgical techniques involved, size of the tendon defect, and duration of graft implantation. Currently, the size of the defect and duration of graft implantation vary between studies and animal models, making it challenging to compare results. Therefore, it would be helpful if guidelines and standards for evaluating graft efficacy in each animal model were established [310].

**Table 8:** Major animal models for tendon repair.

Bridging grafts				
Injury model	Key surgical technique	Implant site and defect size	Duration of graft implantation	References
***Intra-tendinous full-thickness transection***	A full segment of the tendon is removed at the midportion. The graft is implanted between the tendon ends using sutures.	Rat Achilles: 3-6 mm	4-16 weeks	[33,[37], [38], [39], [40],^42^,^46^,^201^,^202^,^210^,^211^,^213^]
		Rabbit extensor digitorum tendon: 1 cm	6 weeks	[34]
		Rabbit Achilles: 1.5-3 cm	12 weeks–13 months	[77,205,206,232]
		Micropig patellar: 2 cm	3 months	[204]
***Intra-tendinous window defect***	A partial rectangular or circular section is removed from the tendon at the midportion. The graft is then sutured into the defect site. Alternatively, sutures are wrapped around the tendon containing the implanted graft to prevent displacement.	Rat patellar: ∼4 mm^2^	2-8 weeks	[41,216,318]
		Rat Achilles: 5 mm^2^	4 weeks	[212,319]
		Rabbit patellar: 54 mm^2^	6 months	[36]
		Rabbit rotator cuff: 50 mm^2^	12 weeks	[35]
**Augmenting grafts**				

**Injury model**	**Key surgical technique**	**Implant site**	**Duration of graft implantation**	**References**

**Full-thickness tendon rupture**	The tendon is cut at the midportion and reconnected with surgical sutures. The graft is then applied over the repaired tendon as a patch or a circumferential wrap.	Mouse superficial flexor digitorum longus tendon	10 days	[45]
		Mouse Achilles tendon	7 days	[214]
		Rat Achilles tendon	3-6 weeks	[44,99,222,267]
		Rabbit flexor digitorum superficialis tendons	3 weeks	[238]
		Rabbit flexor digitorum profundus	3 weeks	[241]
		Rabbit Achilles tendon	3 weeks	[247]
		Rabbit rotator Cuff	6 weeks	[230]
		Chicken flexor digitorum profundus	8 weeks	[246]
		Dog flexor digitorum profundus tendons	9 days	[223]
**Crush injury**	The mid-central area of the tendon is compressed using a 100 g vascular clip for 5 min. The graft is applied over the wound site.	Rat Achilles tendon	4 weeks	[215]

While animal studies can provide preliminary results on the performance of tendon grafts *in vivo*, no animal model can fully recapitulate the injury and repair of human tendons due to key translational differences including size, biology, biomechanics, metabolism, and inflammatory responses [320]. Furthermore, animal injury models do not simulate the usual timing of human graft implantations as they are usually performed soon after injury [40,44]. In contrast, tendon reconstruction surgery in humans is typically performed after the failure of conservative treatment or primary repair, at which point the inflammatory and proliferative stages of tendon healing have already occurred [2].

### 5.3 Measurement of outcomes

The efficacy of implanted grafts is assessed in *situ* and by explant analysis. *In situ* evaluation allows researchers to monitor a broad range of physiological responses including early functional performance of the regenerated tendon, graft integration over time, and inflammation and post-implantation outcomes [321]. Explant evaluation reveals the quality and facilitates the quantification of repair, and regenerated tissue through e.g. histology, molecular, and genomic analyses. Table 9 summarizes procedures used to assess tendon repair *in situ* and following explantation. Prior to the animal studies, power analysis should be conducted to identify the sample size required to detect the reproducible effects of the implanted grafts [322]. Furthermore, random allocation, allocation concealment, and blinding of investigators assessing outcomes are required to reduce bias, particularly in qualitative outcome measures, such as histological analyses [323].

**Table 9:** Outcome measures in animal models of tendon repair.

Analysis methods	Parameters	References
*In situ* evaluation		
High-frequency ultrasound imaging	Monitor tissue swelling at the repair site, graft degradation, and thickness of the repaired tendon	[41]
Magnetic resonance imaging (MRI)	Evaluate the anatomic position of the graft, inflammatory response to the graft, degree of swelling, signal intensity, tissue contours	[40,41]
Gait analysis	Achilles tendon functional test where the size and intensity of the animal's paw prints indicate the extent of recovery	[37,222,321]
TDSC labelling and tracking	To evaluate the recruitment of endogenous cells by grafts, PKH67-labeled TDSCs are injected into the tail vein of the animal after implantation surgery, their distribution within the repair site can be tracked using a non-invasive fluorescence detection system	[37]
*Explant evaluation*		
Scale	Tendon weight and water content	[99]
Macroscopic observation	General appearance	[33,41,99,206]
	Peritendinous adhesion	
	Interface healing between engineered and host tendon	
	Tendon cross-sectional area	
	Nearby muscle size	
Hematoxylin and eosin (H&E) staining	Histomorphometric scoring systems designed for tendon repair evaluation that look at features including •ECM organization of the tendon •Cellularity/cell-matrix ratio •Cell alignment •Cell distribution •Cell nucleus morphology •Organization of repair tissue in the tendon callus •Configuration of callus •Degenerative changes/tissue metaplasia •Inflammation •Cartilage formation •Vascularity	[211,212]
Polarized light microscopy of Sirius red staining	Differentiate between type I and type III collagen	[37,99]
Masson's trichrome staining	Quantify collagen deposition	[47,99,267]
Collagen quantitative assay kit	Quantify collagen deposition	[201]
Multiphoton second harmonic generation (SHG) imaging	Examine collagen density	[41]
Alcian Blue staining	Stain for GAGs, indicating the presence of fibrocartilaginous tissue	[40,267]
Biomechanical analysis	Tensile strength	[99,206,242]
	Young's modulus	
	Strain at failure	
	Failure mode	
	Gliding excursion	
Atomic force microscopy	Nanostructure and nanomechanical properties of the regenerated tendon	[40,213]
Micro-computed tomography (micro-CT)	Density of connected bones which reflect the functional and structural quality of the repaired tendon	[40]
Immunohistochemistry	Tendon-related markers Type I collagen, type III collagen, procollagen type I, tenomodulin, scleraxis, tenascin-C	[37,40]
	Stem cell-related markers	[37,40]
	CD146 and CD44	
	Proliferating cell markers	[45,247]
	Ki-67	
	Bone - related markers (indicating heterotopic ossification)	[40,214]
	RUNX 2, OCN, OCT	
	Cartilage-related markers	
	Type II collagen, SOX9	
	Scar tissue-related markers	[40]
	S100A4, α-SMA, CD68	
	Vascularization	[40]
	CD31 and VEGFR2	
	Inflammation-related	[37,225]
	M1 macrophages – CD68, CD197	
	M2 macrophage – CD163, CD206	
	T-lymphocytes – CD3	
	Pro-inflammatory cytokine	
	IL-1β	
Cytokine multiplex analysis (Luminex assay)	Proinflammatory cytokines	[46]
	IL-1α, IL-1β, IL-2, IL-12, IL-6, TNF-α, IFN-γ, GM-CSF	
	Anti-inflammatory cytokines	
	IL-4, IL-10	
ELISA	Inflammation-related cytokine examination	[37,39]
	Pro-inflammatory – IL-6, IL-1α, VEGF, MCP-1, IL-1β, TNF-α	
	Anti-inflammatory – IL-4, IL-10	
Transmission electron microscope (TEM)	Distribution and diameter of collagen fibrils	[206]
Quantitative PCR	Evaluate the relative expression level of tendon-related genes Type I collagen, type III collagen, tenascin, tenomodulin, fibronectin, biglycan, scleraxis	[33,35,37,77]

**Abbreviation**: α-SMA – alpha-smooth muscle actin; GAG – glycosaminoglycan; GM-CSF – granulocyte macrophage colony stimulating factor; IFN-γ – interferon-γ; IL-1α – interleukin-1α; IL-1β – interleukin 1β; IL-10 – interleukin 10; IL-2 – interleukin-2; IL-12 – interleukin-12; IL-4 – interleukin-4; IL-10 – interleukin-10; IL-6 – interleukin-6; MCP-1 – monocyte chemoattractant protein-1; OCN – Osteocalcin; OCT – Octamer-binding transcription factor; RUNX2 – Runt related transcription factor 2; SOX9 - SRY-box transcription factor 9; TNF-α – tumor necrosis factor-α; VEGF – vascular endothelial growth factor; VEGFR – vascular endothelial growth factor receptor 2.

## 6 Conclusion and outlook

The repair of tendon injuries can be complicated by inferior healing and the development of peritendinous adhesions, which together result in sub-optimal biological and biomechanical properties and an increased risk of re-rupture. Two types of tendon grafts, bridging and augmenting grafts, are commercially available as treatments for enhancing tendon healing, but their efficacies vary, and these are compounded by reports of tendon re-tear and persistent pain. In the past five years, the focus of tissue engineered tendon graft utilization has shifted from bridging to augmenting roles. Through the incorporation of bioactive adjuncts and composite graft structures, recently developed tissue engineered tendon grafts are often multifunctional, with the potential to improve the functional healing of tendons by promoting the recruitment of endogenous tendon cells, modulating the inflammatory response, depositing tendon-specific organized ECM, and lessening fibrotic scars and peritendinous adhesions. The therapeutic efficacy of these grafts benefits from evaluation using animal models, but limitations due to model-dependent outcomes and the choice of measurement methods make it challenging to compare between these studies. Regardless of the constraints, notable outcomes for graft-facilitated tendon repair are seen and include the formation of tendon-specific ECM with appropriate morphology and density, improved mechanical properties that are superior to primary repair, and restored mobility and functionality.

Though tissue engineered tendon grafts have achieved promising therapeutic effects in animal models, the complexities of therapeutic intervention and its timing need to be carefully considered. Many signaling pathways have distinct roles in different tendon healing phases, for example, the TGF-β1/Smad3 signaling pathway is needed to form provisional matrix in the early phase of tendon healing but contributes to scar formation in the later stage. The use of COX inhibitors to suppress inflammation can impede tendon healing in the acute inflammatory phase but has positive effects on the tendon mechanical strength when administered in the remodeling phase. The targeted use of endogenous biomolecules at defined stages of tendon healing should facilitate appropriate temporal and biochemically coordinated tendon repair, accompanied by improved regeneration and enhanced remodeling. There is additional value in investigating the sequence and timing of inflammation by modulating the function of pro-inflammatory and anti-inflammatory macrophages and associated downstream signaling pathways in tendon healing and adhesion formation. Future research should also focus on identifying the mechanical requirements for bridging grafts and draw on predictive modeling. Strategies that simultaneously promote intrinsic healing while inhibiting extrinsic healing, and encourage regeneration of compartmentalized structure should continue to be developed.

**Key points**

• Inferior healing outcomes and peritendinous adhesions remain clinical challenges in tendon repair.
• Tendon grafts used to facilitate tendon repair and reconstruction are generally constructed as either bridging or augmenting grafts.
• Current research on tendon graft functionality focuses on promoting a balance between intrinsic and extrinsic healing.
• Incorporation of bioactive adjuncts into tendon grafts can lead to endogenous tendon cell recruitment, modulation of the inflammatory response, deposition of tendon-specific organized ECM, and a reduction in fibrotic scarring and peritendinous adhesions.
• Assessment of the therapeutic efficacy of tendon grafts benefits from evaluation using animal models, but model-dependent outcomes and the variety of measurement methods make it challenging to make comparisons between studies.

**CRediT authorship contribution statement**

**Miao Zhang:** Writing – original draft. **Suzanne M. Mithieux:** Writing – review & editing. **Ziyu Wang:** Writing – review & editing. **Anthony S. Weiss:** Writing – review & editing.

## Declaration of competing interest

The authors declare that they have no known competing financial interests or personal relationships that could have appeared to influence the work reported in this paper.

## Data Availability

No data was used for the research described in the article.
